# Partners in plasticity: serotonergic glial interactions in brain circuit remodeling

**DOI:** 10.3389/fnins.2026.1782246

**Published:** 2026-04-10

**Authors:** Vanessa Kay Miller, Kendal Broadie

**Affiliations:** 1 Department of Biological Sciences, Vanderbilt University and Medical Center, Nashville, TN, United States; 2 Department of Cell and Developmental Biology, Vanderbilt University and Medical Center, Nashville, TN, United States; 3 Department of Pharmacology, Vanderbilt University and Medical Center, Nashville, TN, United States; 4 Kennedy Center for Research on Human Development, Vanderbilt University and Medical Center, Nashville, TN, United States; 5 Vanderbilt Brain Institute, Vanderbilt University and Medical Center, Nashville, TN, United States

**Keywords:** 5-HT, 5-HT2A receptor activation, brain circuit remodeling, experience-dependent brain circuit remodeling, extracellular matrix remodeling, Fragile X syndrome, glia, glial 5-HT biosynthesis

## Abstract

Experience-dependent brain circuit optimization choreographed by environmental sensory input activity involves synapse formation, pruning, and remodeling to sculpt appropriate connectivity. The serotonin (5-HT) neuromodulator acts as a core regulator of this circuit plasticity. Classically, serotonergic control has been understood solely through neuronal mechanisms, however new evidence reveals glial 5-HT signaling roles. This review focuses on recent studies in *Drosophila* with reference to foundational mammalian work to discuss 5-HT functions in both neurons and glia, particularly experience-dependent extracellular matrix remodeling, glial infiltration, and synapse elimination in early-life critical periods. Disruption of serotonergic regulation is proposed to contribute to a spectrum of neurodevelopmental disorders, including Fragile X syndrome, in which failure to prune and persistence of immature connectivity cause severe life-long behavioral impairments. Recent discoveries further reveal targeted induction of glial serotonergic signaling can re-open “critical period-like” synapse pruning at maturity. Enabling large-scale connectivity changes has broad potential therapeutic applications for disease, injury, trauma, and cognitive dysfunction. A key advance is the emerging evidence that glia—not just neurons—are serotonergic mediators of synaptic remodeling: glial 5-HT biosynthesis, 5-HT_2A_ receptor activation, and matrix metalloprotease-mediated function together allow access for experience-driven synapse elimination. We propose glia-to-glia class serotonergic signaling—linking sensory experience to synapse pruning—may represent a conserved plasticity gating mechanism that determines whether circuitry is permissive or resistant to synaptic connectivity modification. Harnessing glial class-specific serotonergic control of experience-dependent brain circuit remodeling may enable new targeted therapies to correct brain function while avoiding the negative side effects of global serotonin elevation.

## Introduction

Neural circuit maturation is orchestrated by early genetic programming followed by later activity- and experience-dependent mechanisms that refine connectivity in response to environmental input (Katz and Shatz, 1996). Foundational studies first established this progression, notably work in visual and somatosensory systems demonstrating early-life sensory deprivation disrupts synapse connectivity refinement, critical period timing, and the emergence of mature behavioral outputs (Hubel and Wiesel, 1970; Wagor et al., 1980). Over the past decade, cutting-edge approaches—including multiomics, cell-type specific manipulations, and *in vivo* Ca^2+^ imaging—have revealed how early sensory experience drives transcriptomic shifts, glia-mediated synapse remodeling, and network-level reorganization that collectively shape circuit architecture in mammalian models (Kalambogias et al., 2019; Heffel et al., 2024; Parkins et al., 2025). Decades of work on vision, audition, and somatosensation shows patterned sensory activity governs synaptogenesis, synapse pruning, and dendritic remodeling to achieve precise connectivity (Peter, 1979; Knudsen, 1983; Pons et al., 1991). More recent studies reveal molecular mechanisms underlying this refinement, identifying neuromodulator gating, extracellular matrix dynamics, and phagocytic pathways as key determinants of experience-dependent plasticity (Pizzorusso et al., 2002; Froemke, 2015; Hong et al., 2016a). Building on these mammalian studies, *Drosophila* emerged as a powerful genetic system for mechanistic discovery, revealing with single-cell and synapse-level precision how sensory input shapes neural circuit remodeling across visual, olfactory, and mechanosensory systems (Sugie et al., 2015; Golovin and Broadie, 2016; Golovin et al., 2019; Carreira-Rosario et al., 2021). With sophisticated genetic and connectomic tools, *Drosophila* provides means to reveal causal links between experience, molecular effectors, and lasting outcomes in circuit remodeling, offering a tractable system to discover conserved principles of experience-driven brain circuit development and to identify novel mechanisms enabling critical period plasticity (Schlegel et al., 2024).

The neuromodulator serotonin (5-HT) has long been recognized for its key conserved roles, with seminal mammalian studies revealing widespread synaptic plasticity functions (Jacobs and Fornal, 1999; Cavaccini et al., 2018). Serotonin modulates mature circuit function, but is also central in early life to shaping neural circuit assembly, with alterations in 5-HT signaling disrupting dendritic growth, synaptic refinement, and critical period timing in sensory and associative brain regions (Gaspar et al., 2003; Ansorge et al., 2004; Miceli et al., 2013). Importantly, disruptions in serotonin signaling have been repeatedly linked to neurodevelopmental circuit disorders, including autism spectrum disorders and intellectual disabilities, with Fragile X syndrome (FXS) providing a particularly compelling example of serotonergic dysfunction aligning with failures in synaptic refinement, critical period closure, and long-term behavioral impairments (Doll and Broadie, 2015, 2016; Doll et al., 2017). Recent breakthroughs in mammalian research—leveraging glia-targeted manipulations, receptor-specific pharmacology, and *in vivo* imaging—show that serotonin is a potent signal orchestrating cytoskeletal remodeling, synaptogenesis, and activity-dependent synapse pruning to sculpt circuit connectivity (Albertini et al., 2023; Ogelman et al., 2024). In parallel, *Drosophila* has emerged as a powerful genetic model for mechanistically dissecting serotonin-dependent remodeling, with single-cell precision, synapse-resolution connectomics, and temporally-controlled activity tools revealing causal links between serotonergic signaling, circuit refinement, and behavioral outcomes (Bonanno and Krantz, 2023; Dopp et al., 2024; Dorkenwald et al., 2024). Moreover, the *Drosophila* FXS disease model demonstrates direct manipulation of serotonin, 5-HT receptors, and downstream effectors can restore defective circuit maturation and behavioral impairments, establishing a platform for uncovering serotonergic mechanisms of neurodevelopmental disorders (Tessier and Broadie, 2008; Sears and Broadie, 2018; Song and Broadie, 2023).

Re-opening experience-dependent circuit remodeling in the adult brain represents a compelling challenge, given the capacity for large-scale synaptic remodeling dramatically declines after early life (Patton et al., 2019; Shang and Zhang, 2025). Foundational mammalian studies in sensory-driven circuit refinement—particularly in visual and somatosensory systems—established critical periods as transient windows in which patterned sensory experience drives synaptogenesis, synapse pruning, and circuit remodeling to optimize mature circuit function (Hubel and Wiesel, 1970; Zuo et al., 2005; Briner et al., 2010). For decades, critical period closure was considered irreversible, yet recent work employing molecular, pharmacological, and behavioral paradigms demonstrates that adult circuits retain a latent plasticity capacity that can be reactivated under specific conditions, allowing experience-dependent remodeling on a scale once thought restricted to critical periods (Patton et al., 2019). Harnessing this potential has profound implications for repairing injured or dysregulated circuitry, with therapeutic relevance extending from cortical recovery after stroke and traumatic injury, to maladaptive memory reconsolidation in post-traumatic stress disorder, to sensory and cognitive disruptions in schizophrenia, and—most critically in the context of neurodevelopmental disorders—to lifelong connectivity impairment rooted within aberrant critical period mechanisms (Astill Wright et al., 2021; Hordacre et al., 2021; Vinogradov et al., 2023). Emerging evidence suggests that serotonergic interventions, including psychedelic and other 5-HT-targeted drugs, may enable the re-opening of “critical period-like” states in adulthood by alleviating constraints on circuit remodeling, allowing large-scale synapse connectivity modulation and enhancing experience-driven learning (Grieco et al., 2022; Weiss et al., 2025). These advances support the rapidly-growing suggestion that serotonin is not only a developmental regulator of circuit remodeling, but may also be a powerful lever for reinstating experience-dependent plasticity in the mature brain, offering a potential transformative path toward correcting brain circuit dysfunction.

Although neuronal functions have long been the primary focus of 5-HT-dependent modulation, accumulating evidence now reveals a crucial role for serotonergic glia in shaping neural circuit synaptic connectivity (Miller and Broadie, 2024, 2025; Duffy and Eyo, 2025; Nelson et al., 2025). Although long dismissed as passive support cells, foundational mammalian studies demonstrated glia actively participate in both synapse formation and pruning (Christopherson et al., 2005; Paolicelli et al., 2011). Indeed, multiple classes of glial cells, including radial glia, astrocytes, and microglia, have essential roles in synapse remodeling. Acting as phagocytes, multiple glial classes eliminate excess or inappropriate synapses to sculpt maturing circuits (Neniskyte and Gross, 2017; Raiders et al., 2021). Similar mechanisms are conserved in the *Drosophila* model. One class of *Drosophila* phagocytic glia, the ensheathing glia (EG), are experience activated, infiltrate neuropil, and mediate contact-mediated recognition resulting in targeted synapse elimination (Vilalta and Brown, 2018). *Drosophila* astrocyte-like glia (ALG) regulate both the formation and pruning of synapses in response to activity-dependent neuronal Ca^2+^ signaling (Vilalta and Brown, 2018; Bajar et al., 2022). More recently, mouse studies have revealed that glia express serotonin transporters and receptors, with serotonergic signaling directly modulating glial phagocytic activity during development (Kolodziejczak et al., 2015). Similarly in *Drosophila*, glial serotonin production and glial 5-HT_2A_ receptor signaling have been recently found to be essential for experience-dependent synaptic pruning during a juvenile critical period (Miller and Broadie, 2024). This dual glial function reveals a novel glia-to-glia signaling mechanism with serotonin production required only in ensheathing glia and 5-HT_2A_ receptors required only in astrocyte-like glia (Miller and Broadie, 2025). Through this glial serotonergic signaling, glial phagocytes actively sculpt experience-driven synaptic connectivity in a sensory level-dependent, temporally-restricted critical period mechanism—fundamentally redefining the cellular means by which serotonergic modulation can govern brain circuit remodeling.

This review discusses the emerging framework of serotonergic signaling functions—traditionally based solely on neurons, but here expanded to include glia—in the regulation of experience-dependent neural circuit remodeling, with broad mechanistic roles but highlighting glial phagocyte synapse pruning (Miller and Broadie, 2024; Nelson et al., 2024, 2025). We begin with an introduction to serotonin signaling and foundational roles and sources of serotonin in early development. Followed by a brief outline of activity-experience-dependent remodeling, tracing foundational mammalian work on critical periods through to recent advances on molecular, cellular, and systems-level plasticity determinants in *Drosophila* (Hubel and Wiesel, 1970; Golovin et al., 2019; Baumann et al., 2024). We next focus on serotonergic developmental modulation, from earlier work on 5-HT receptor-mediated growth cone dynamics and dendritic maturation, to newer studies on 5-HT signaling orchestration of transcriptomic state transition, ECM remodeling, and synapse pruning (Gaspar et al., 2003; Ogelman et al., 2024). We then move from serotonergic neurons to serotonergic glia, discussing the rapidly expanding appreciation that glia serve as essential effectors of circuit refinement via experience-dependent protease release, synapse phagocytosis, and connectivity remodeling, especially within critical periods (Albertini et al., 2023; Miller and Broadie, 2024; Nelson et al., 2025). Next, we evaluate how disruptions in serotonergic plasticity may contribute to neurodevelopmental disorders, highlighting Fragile X syndrome (FXS) as an example (Golovin and Broadie, 2017; Golovin et al., 2021; Song and Broadie, 2022). Finally, we discuss the therapeutic frontier of re-opening serotonergic plasticity in the adult brain, summarizing evidence that serotonergic psychedelics and 5-HT receptor drugs might reinstate critical period capacities to correct maladaptive wiring following injury, trauma, and disease (Ly et al., 2018; Patton et al., 2019; Astill Wright et al., 2021; Miller and Broadie, 2024, 2025). The major message of this review is that serotonin appears to act as a developmental and activity-dependent neuromodulator of circuit remodeling across the lifespan, operating via both neurons and glia to refine and potentially restore neural connectivity.

## Developmental serotonin neuromodulator signaling

**Figure 1:**
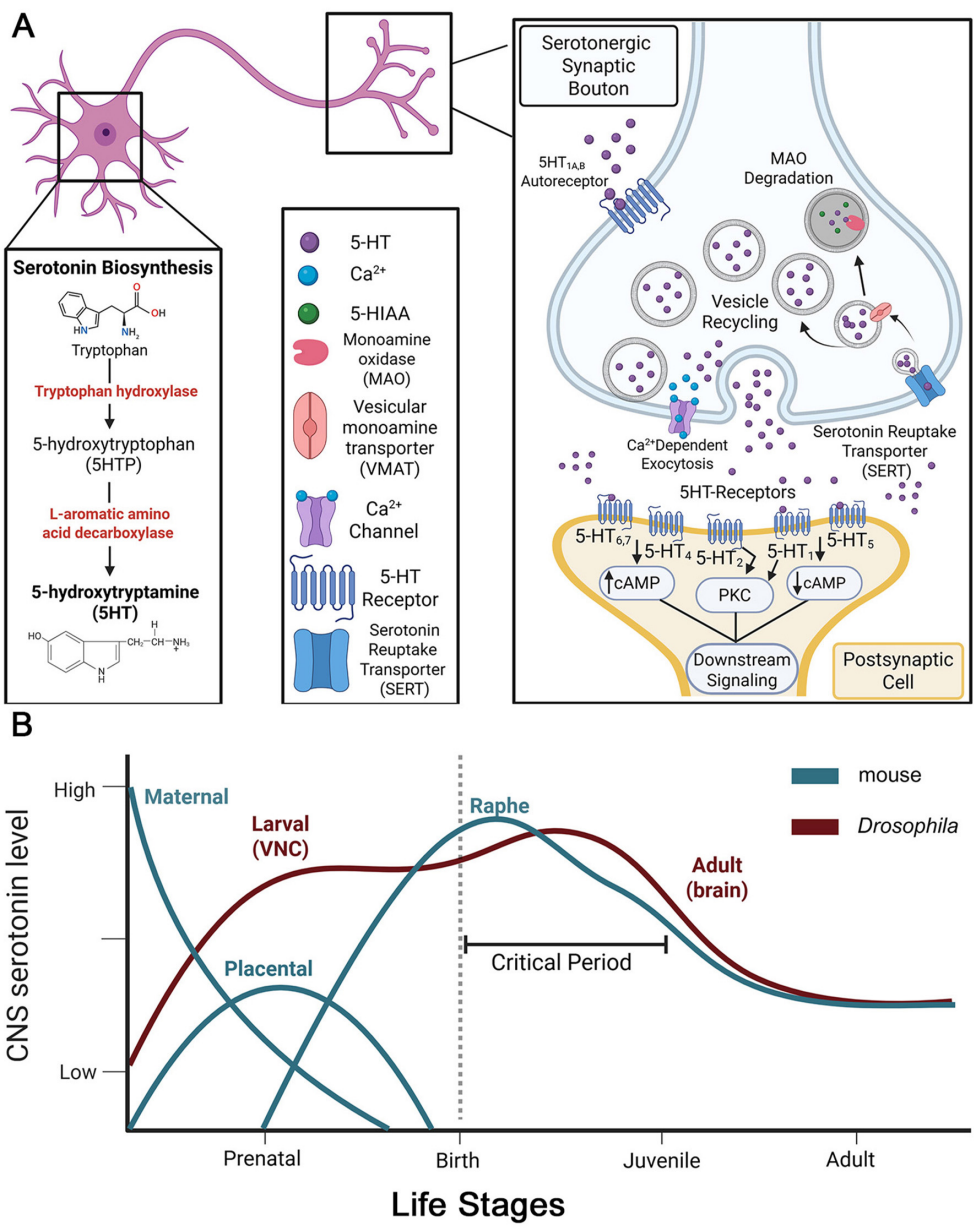
Neuronal serotonergic signaling machinery and 5-HT levels throughout development. **(A)** Schematic of serotonin biosynthesis and synaptic signaling. Left, enzymatic steps in the conversion of tryptophan to 5-hydroxytryptamine (5-HT), beginning with hydroxylation of tryptophan by tryptophan hydroxylase to produce 5-hydroxy-L-tryptophan (5-HTP), followed by decarboxylation by L-aromatic amino acid decarboxylase to generate 5-HT. Middle, molecular components involved in serotonergic signaling, including 5-HT, Ca^2+^, monoamine oxidase (MAO), 5-hydroxyindoleacetic acid (5-HIAA), vesicular monoamine transporter (VMAT), Ca^2+^ channel, 5-HT receptor, and the serotonin reuptake transporter (SERT). Right, schematic of a serotonergic synaptic bouton highlighting Ca^2+^-dependent vesicular exocytosis of 5-HT, vesicle recycling, degradation of excess 5-HT by MAO, and reuptake via SERT. Presynaptic 5-HT autoreceptor subtypes (e.g., 5-HT_1A, B_) sense serotonergic neurotransmission. Postsynaptic 5-HT receptor subtypes (5-HT_1 − 7_) trigger multiple second messenger intracellular signaling cascades; G_s_-coupled receptors increase cAMP, G_i/o_-coupled receptors decrease cAMP, G_q_-coupled receptors activate PKC. **(B)** Schematic of central nervous system 5-HT levels across the lifespan in mice and *Drosophila*, adapted from Carvajal-Oliveros and Campusano (2021). In mice (teal), CNS 5-HT levels are initially dominated by maternal and placental contributions during prenatal development, followed by a large rise in raphe-derived serotonin peaking after birth, coincident with opening of sensory-driven critical periods. Serotonin levels decline following critical period closure and stabilize in adulthood. In *Drosophila* (maroon), embryonic and larval ventral nerve cord (VNC) 5-HT production rises during development, transitioning to raphe-analogous 5-HT sources. 5-HT levels peak during juvenile adult critical period stages, and then decrease into adulthood. Across species, elevated 5-HT levels coincide with experience-dependent remodeling during early-life juvenile critical periods, suggesting conserved serotonergic regulation of neural circuit refinement.

Serotonin is a key neuromodulator for regulating mood, sleep, and appetite (Berger et al., 2009). 5-hyrdroxytryptamine (5-HT) synthesis begins with the amino acid tryptophan, hydroxylated via the tryptophan hydroxylase enzyme to the 5-hydroxy-L-tryptophan (5-HTP) intermediate, which is further decarboxylated by L-aromatic amino acid decarboxylase to make 5-HT (Figure 1A; Salerno et al., 1984; Ebadi and Simonneaux, 1991). Serotonergic cells are specialized via the capacity to turn tryptophan into 5-HT, specifically via the action the rate-limiting tryptophan hydroxylase (Trhn) enzyme (Figure 1A; Noguchi et al., 1973). The conversion of tryptophan to serotonin accounts for only ~5% of tryptophan metabolism, due to the tight restriction of tryptophan hydroxylase to the brain, enterochromaffin cells and, to a much lesser extent, platelets (Hofto et al., 2009). Within the central nervous system (CNS), serotonin is believed to be synthesized and stored primarily in serotonergic neurons (Figure 1A), but also is present in the pineal gland and certain catecholaminergic neurons. Mammalian serotonergic cells are organized into nine discrete clusters localized to the pons and midbrain. The raphe nuclei constitute the principal serotonergic centers (Törk, 1990), giving rise to ascending projections to the forebrain and descending projections to the medulla and spinal cord. Additional, smaller serotonergic nuclei within the medullary reticular formation project locally within the medulla (Fuxe, 1965). Serotonergic neuronal synapses are further characterized by the production of serotonin through presynaptic biosynthesis, 5-HT reuptake via the serotonin reuptake transporter (SERT), and both pre- and post-synaptic 5-HT receptors (Figure 1A, right; Berger et al., 2009). Presynaptic serotonergic signaling begins with 5-HT biosynthesis, with 5-HT then translocated into small clear synaptic vesicles (SVs) or large dense-core vesicles (LDCVs; Tamir et al., 1996). Serotonergic SVs near presynaptic active zones act in rapid signal propagation (Figure 1A, right; Tyler and Murthy, 2004). LDCVs loaded with 5-HT and directive peptides (e.g., Substance P) in the bouton interior require stronger, more prolonged stimulation, and activate extra-synaptic receptors over longer distances (Özçete et al., 2024).

5-HT released into the synaptic cleft localizes signaling to a postsynaptic partner (Figure 1A, right), or via volume transmission to many diffuse targets (Tyler and Murthy, 2004; Ligneul and Mainen, 2023). 5-HT is taken back into cells via the serotonin reuptake transporter (SERT), a Na^+^-dependent 12-pass transporter controlling serotonin signaling capacity (Sneddon, 1973). Presynaptic SERT recycles 5-HT to be reused for synaptic release, or degraded by monoamine oxidase (MAO; Figure 1A, right; Mcisaac and Page, 1959). SERT is also present in other neurons and glia for additional mechanisms to terminate signaling (González-Arias et al., 2023). A defining feature of serotonergic signaling is auto-reception to detect local 5-HT levels and direct presynaptic serotonergic signaling, primarily via 5-HT_1A, B_ receptors (Figure 1A, right; Slifirski et al., 2021). 5-HT_1A_ receptors control serotonin output from the dorsal raphe nucleus, while 5-HT_1B_ receptors regulate local release and influence serotonin reuptake and synthesis (Belmer et al., 2018). Recent studies also implicate 5-HT_2A_ as an auto-receptor in long-term propagation during overexpression by psychedelic activation (Cameron et al., 2023). Postsynaptic propagation of serotonergic signaling occurs via diverse 5-HT receptors (Figure 1A, right). There are 13 G-protein coupled receptors (GPCRs) and 1 ligand-gated ion channel, which are further divided into 5-HT_1 − 7_ classes (Hoyer and Martin, 1997). 5-HT_1A − F_ are G_i/o_ effectors that upon activation also release Gβγ subunits to mediate downstream signaling, such as opening of GIRK potassium channels, inhibition of voltage-gated Ca^2+^ channels, activation of phospholipase C (PLC) and type 2 adenylyl cyclase, whereas the 5-HT_4 − 7_ receptors are G_s_ effectors, which regulate cAMP signaling (Figure 1A, right; Gerachshenko et al., 2005; Guseva et al., 2014; Albert and Vahid-Ansari, 2019). 5-HT_2A − C_ are G_q_ effectors which drive PLC signaling (Hoyer et al., 2002). This expansive receptor diversity expands signaling from a single ligand to multiple different pathways. The 5-HT_2A_ receptor has long been associated with plasticity, and is a primary target for antipsychotic (antagonist) and psychedelic (agonist) drugs (Hoyer et al., 2002). 5-HT_2A_ receptor activation triggers PLC/PKC and MAPK/ERK pathways that upregulate intracellular Ca^2+^ (Figure 1A, right; Masson et al., 2012; Agahari and Stricker, 2021). Importantly, glia (astrocytes and microglia) also express 5-HT_2A_ receptors and elevate intracellular Ca^2+^ via PLC signaling (Glebov et al., 2015; Verkhratsky et al., 2021).

Serotonin plays essential roles in nervous system development, with 5-HT contributions from both the mother and placenta prior to later embryonic 5-HT production (Figure 1B; Carvajal-Oliveros and Campusano, 2021). Before serotonin acts classically as a neuromodulator (Figure 1A), maternal 5-HT acts in the fetal brain as a hormonal/growth/differentiation factor (Sundström et al., 1993). Maternal and placental 5-HT play essential roles in progenitor cell identity as the serotonin signaling drives its own circuitry development (Figure 1B). At early stages of mouse development, as early as neural tube formation, the developing embryo cannot make serotonin and high 5-HT levels come from maternal and placental origins (Bonnin and Levitt, 2011). In *Drosophila*, 5-HT is produced in early embryonic stages, where it is required for germband extension (Figure 1B). Similar to mammals, peak 5-HT synthesis at the beginning of germband extension is strictly dependent upon the maternal deposition of biopterins, products of GTP-cyclohydrolase and cofactors of tryptophan hydroxylase, and then later upon zygotic synthesis of both tryptophan hydroxylase and DOPA decarboxylase enzymes (Colas et al., 1999). In mice, serotonergic neurons are evident as early as 5 days gestation, and by 15 days the raphe nuclei contain a typical arrangement of a serotonergic circuit (Figure 1B; Sundström et al., 1993; Carvajal-Oliveros and Campusano, 2021; Wegiel et al., 2024). Serotonin tropic roles are distinct during early stages of development compared to serotonin neuromodulatory roles in adults, which are controlled by neurotransmission activated locally within neural circuitry to control mood, appetite, memory, and cognition (Rosenfeld, 2019; Pourhamzeh et al., 2021). Early post-natal development is more complex, with less clear distinctions between serotonin roles as a tropic growth factor and neuromodulator (Brummelte et al., 2016). Postnatal plasticity relies on heightened serotonergic signaling (Figure 1B), for example in the refinement of axon terminal arborization in response to the environmental signals (Bonnin and Levitt, 2011). Thus, birth (in mice) or eclosion (in *Drosophila*) represent an important transition to peak serotonin levels that regulate the juvenile experience-dependent remodeling of the nervous system.

Experience-driven critical period neural circuit refinement, specifically synapse elimination, involves heightened levels of serotonergic signaling (Figure 1B). The temporally-transient critical period windows of remodeling permit both large-scale synapse formation and elimination (Holtmaat and Svoboda, 2009), with critical periods overall characterized by a net loss of synapses at closure (Bourgeois et al., 1994). Serotonergic signaling disruptions in structural remodeling during sensitive developmental windows cause permanent impairments in adults (Gaspar et al., 2003). In mammals, the dorsal raphe is the principal source of brain serotonin, and its maturation underlies a well-defined postnatal “5-HT critical period” during which serotonin levels are transiently elevated and early experience strongly sculpts circuit wiring (Figure 1B). In mice, several studies identify heightened sensitivity to serotonergic manipulations across the first postnatal week (P2–P11), with region-specific peaks in serotonergic innervation/5-HT content during this interval of postnatal development, consistent with a short, high 5-HT critical window influencing cortical and subcortical circuit maturation (Figure 1B; Rebello et al., 2014; Brummelte et al., 2016). Importantly, mouse embryonic/fetal 5-HT production can arise not only from nascent raphe neurons but also from transient maternal/placental sources that supply serotonin to the forebrain during initial circuit formation, linking these peripheral sources to early signaling (Figure 1B; Bonnin and Levitt, 2011). In comparison, *Drosophila* serotonergic neurons arise early in embryogenesis and post-hatching larvae show heightened, juvenile-stage serotonin dynamics (measured electrically and by real-time assays) during a period of sensory experience-dependent remodeling (Figure 1B; Sykes and Condron, 2005). While both mice and *Drosophila* employ transiently elevated 5-HT to gate heightened plasticity, mice rely on placental/fetal and raphe-derived 5-HT pools with postnatal peaks tied to mammalian cortical maturation, whereas *Drosophila* depend on intrinsically produced central ventral nerve cord (VNC; Figure 1B) serotonin during larval/juvenile stages, and exhibit distinct temporal scaling within early critical periods (Sykes and Condron, 2005).

Glia are also intimately involved in serotonin signaling. During initial prenatal development, serotonin promotes astrocyte proliferation, where it influences early differentiation (Whitaker-Azmitia et al., 1990; Whitaker-Azmitia, 2001). Astrocytic 5-HT signaling promotes excitatory synapse formation in mice (Figure 1B; Müller et al., 2021), with serotonin also indirectly regulating neural circuit maturation through glial pathways (Verkhratsky et al., 2021). Mouse microglia utilize 5-HT_2B_ receptors to modulate synaptic pruning and blocking these 5-HT_2B_ receptors disrupts microglia-mediated synaptic refinement in the juvenile postnatal cortex (Kolodziejczak et al., 2015). In *Drosophila*, glial serotonin production plays a central, essential role in the juvenile adult olfactory critical period remodeling and is localized to specific glial classes that drive targeted synapse elimination (Figure 1B; Miller and Broadie, 2024). Recent work shows that glia—not neurons—are the cells required to both synthesize serotonin and express 5-HT_2A_ receptors during this critical period, and that glial 5-HT production and glia-to-glia serotonergic signaling is required for experience-dependent pruning of synaptic glomerular innervation. Moreover, conditional institution of glial serotonergic signaling in mature adults, when 5-HT levels are low (Figure 1B) can re-open “critical period-like” pruning, indicating glial serotonergic signaling is limiting to this normally transient plasticity (Miller and Broadie, 2024). More recent work on this glia-to-glia serotonergic signaling shows it triggers downstream matrix metalloproteinase (MMP) induction to enable glial infiltration and phagocytic pruning of synapses (Miller and Broadie, 2025), showing that glial 5-HT synthesis is causally tied to experience-dependent remodeling (Figure 1B). These very recent findings complement older *Drosophila* critical period studies of juvenile temporally-restricted timing in olfactory experience-driven synapse pruning (Golovin et al., 2019), to place glial 5-HT production at the center of this critical period plasticity (Figure 1B). Overall, glial serotonergic neuromodulation is essential to gate downstream mechanisms driving neural circuit connectivity remodeling.

## Serotonin signaling in critical period circuit remodeling

**Figure 2:**
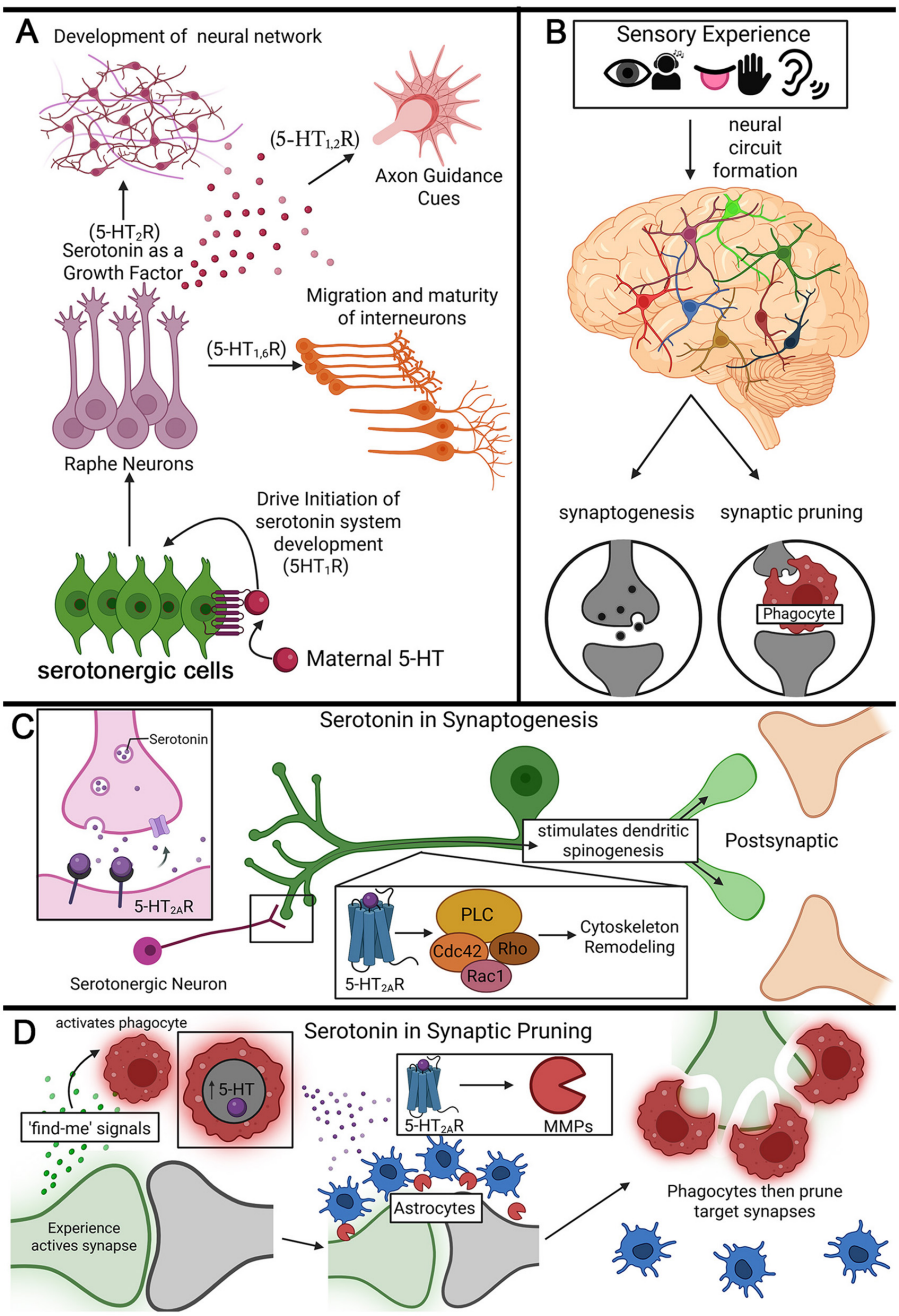
Serotonin signaling roles across multiple stages on nervous system development. **(A)** Early developmental roles of serotonergic signaling in establishing the nervous system. Maternally derived 5-HT acts on serotonergic progenitors via 5-HT_1_ receptors to initiate the formation of the serotonergic circuitry. As development proceeds, 5-HT functions as a growth and differentiation factor, promoting axon growth cone guidance through 5-HT_1, 2_ receptors, and supporting the migration and maturation of interneurons through 5-HT_1, 6_ receptors. Elevation of 5-HT from newly-born raphe neurons subsequently drives the assembly of distributed serotonergic neural networks. **(B)** Postnatal sensory experience shapes circuit architecture through activity-dependent remodeling. Sensory input triggers neuronal network refinement by coordinating synaptogenesis (formation of new synapses) and synaptic pruning (removal of inappropriate or weak synapses), which together optimize the circuit connectivity during early-life critical periods. **(C)** Serotonin-dependent mechanisms of synaptogenesis. Serotonergic neurons release 5-HT onto postsynaptic targets where 5-HT_2A_ receptor activation drives phospholipase C (PLC) signaling and regulation of downstream actin cytoskeletal regulators (Cdc42, Rho, Rac1), stimulating dendritic spinogenesis and growth cone remodeling to promote *de novo* synapse formation. **(D)** Model of serotonin-dependent mechanisms of synaptic pruning from *Drosophila* studies. Sensory experience induces the release of “find-me” guidance cues from target synapses, elevating glial 5-HT levels and infiltration. In astrocytes, 5-HT_2A_ receptor signaling drives matrix metalloprotease (MMP) production for localized extracellular matrix (ECM) degradation, granting glial phagocytes access to targeted synapses. This coordinated glial astrocyte-to-phagocyte signaling enables selective, experience-dependent synapse elimination.

During prenatal development, serotonin works as a neurotropic factor that modulates progenitor cell proliferation, neuronal migration, and axonal wiring (Figure 2A; Brummelte et al., 2016). Much like other neuromodulators during early development, serotonin is released via large dense-core vesicles and is proposed to act as a morphogen-like gradient signal to drive progenitor development (Figure 2A). In particular, early serotonin signaling promotes progenitor differentiation for the serotonergic circuitry, including raphe neurons (Azmitia, 2001). Maternally-derived 5-HT is recognized by progenitors through 5-HT_1_ receptors, which propagate signaling cascades to form the raphe nuclei clusters of serotonergic neurons (Figure 2A; Bonnin and Levitt, 2011; Bonnin et al., 2011). Subsequently, 5-HT_1_ and 5-HT_6_ receptors drive cellular migration and maturity of interneurons within localized networks around their dendritic arbors (Patel and Zhou, 2005; Chaumont-Dubel et al., 2020). Both 5-HT_1_ and 5-HT_2_ receptors function in growth factor signaling that drives axonal guidance and later synaptogenesis leading to the development of serotonergic system (Figure 2A; Vicenzi et al., 2020). In mammals, the prefrontal cortex epicenter of higher-order cognition is built on the foundation for this circuit formation (Chen et al., 2025). Serotonergic axons originating from the raphe nuclei densely innervate the prefrontal cortex and modulate the assembly of higher-order cognition circuits (Figure 2A; Ogelman et al., 2024). Serotonin signaling disruptions in early development result in behavioral impairments that last into adulthood (Fabio et al., 2025). One example from mice is that elevated serotonin levels in the prefrontal cortex from early prenatal exposure to serotonin reuptake inhibitors (SSRIs) via maternal/placental blood is associated with behavioral impairments such as anxiety, depression, and maladaptive stress responses throughout later life (Ansorge et al., 2004; Bonnin et al., 2011). This early developmental serotonergic signaling circuitry formation laying the foundations for lifelong behavioral states is driven solely by internal genetic factors and appears independent of external experience influences.

Serotonin plays a central role in postnatal experience-dependent refinement of the neural circuits in juveniles (Figure 2B). Sensory critical periods are defined windows of time following birth that begin with the onset of sensory experience and end with closure caused by forces resistant to further circuit remodeling (Hensch, 2005; Reha et al., 2020). In response to sensory input, neural circuits are sculpted through a dynamic balance of synapse formation and elimination (Figure 2B; Hubel and Wiesel, 1970; Antonini and Stryker, 1993; Coulson et al., 2022; Larsen et al., 2023). In the *Drosophila* early-life olfactory critical period, serotonin production is strongly upregulated in circuit-localized signaling domains in response to appropriate odorant input sensory experience (Miller and Broadie, 2024). For example, exposure to the odorant ethyl butyrate (EB) during the critical period, and only during the critical period, elevates serotonin levels surrounding the EB-responsive VM7 olfactory sensory neuron synaptic glomeruli within the juvenile brain. The same experience at maturity causes no changes in serotonin signaling (Miller and Broadie, 2024). Heightened EB experience results in synaptic pruning (Figure 2B), in a transient, dose-dependent, and reversible mechanism during this critical period (Golovin and Broadie, 2016; Golovin et al., 2019). When the ability to experience this odorant experience is blocked, with cell-targeted genetic manipulations, both the circuit-localized serotonin upregulation and targeted experience-dependent synapse pruning is likewise completely blocked (Miller and Broadie, 2024). This work establishes a direct mechanistic relationship between critical period experience, local serotonergic signaling, and synapse elimination (Figure 2B). Serotonergic signaling also plays important roles in the opposing direction of synaptogenesis (Higa et al., 2022, 2024; Udoh et al., 2024). In the experience-dependent formation of new synapses, serotonin acts as a signal to stimulate dendritic spinogenesis (Figure 2C; Jones et al., 2009; Rojas et al., 2017). Thus, serotonergic signal timing, level, and the receptor-mediated responses must determine the directionality of circuit remodeling.

Presynaptically, during an early critical period in mammalian models (~2–3 weeks postnatal), serotonin modulates glutamate release via 5-HT_2_ receptors to drive preferred recurrent excitatory connectivity in the neocortex (Agahari and Stricker, 2025). Serotonin signaling also induces excitatory postsynaptic potentials in apical dendrite via glutamatergic modulation in neocortical pyramidal cells (Aghajanian and Marek, 1997). This glutamatergic modulation via serotonin contributes to *de novo* growth of functional spines in the developing cortex, with newly-formed spines induced by spatially precise activity becoming rapidly functional and incorporated into cortical circuits (Kwon and Sabatini, 2011). Such serotonergic modulation in neuron-to-neuron signaling is specific to subsets of cortical neuron spines via 5-HT_2A_ receptors (Jones et al., 2009) Further, serotonergic modulation at presynaptic sites can increase spontaneous glutamate release; however, not all pyramidal cells receive these inputs, indicating synapse-specific effects in which serotonin selectively maintains or modulates certain dendritic spines (Agahari and Stricker, 2021, 2025). Psilocybin, a serotonergic psychedelic, increases the density of dendritic spines in pyramidal tract (PT) and intratelencephalic (IT) neurons in the medial prefrontal cortex, but exerts its ability to ameliorate stress-related defects through PT neurons only, demonstrating that psilocybin is capable of strengthening specific sensory (presynaptic) input to PT neurons. Taken together, these studies indicate that this serotonin receptor agonist is capable of inducing postsynaptic (dendritic spine) structural remodeling and altering presynaptic input strength in specific manner within select neurons (Shao et al., 2025; Jiang et al., 2026). Serotonergic neurons also synapse onto the dendrites of postsynaptic target cells and via activation of the 5-HT_2A_ receptor trigger a PLC signaling pathway to drive actin cytoskeleton remodeling (Figure 2C; Ohtani et al., 2014; Vicenzi et al., 2020). 5-HT_2A_ receptor-dependent PLC activation phosphorylates Cdc42, Rac1, and Rho, which all play coordinated signaling roles (Jones et al., 2009; Dai et al., 2010; Mi et al., 2017). The actin cytoskeleton remodeling is essential for the motility of neuronal growth cones and subsequent formation of new synapses (Figure 2C; Penzes and Rafalovich, 2012; Lei et al., 2016). Thus, the role of serotonin in synaptogenesis involves a neuron-to-neuron signaling mechanism. However, extensive studies on glial roles in synapse formation suggest glial mechanisms (Daubert and Condron, 2010; Shan et al., 2021; Farizatto and Baldwin, 2023). In experience-dependent pruning of presynaptic boutons, a major glial serotonergic signaling role has emerged (Figure 2D; Miller and Broadie, 2024). This synapse elimination is based on structural connectivity changes only, with no electrophysiological or calcium imaging studies of synaptic function. In the *Drosophila* olfactory critical period, sensory experience activates phagocytic glia which upregulate 5-HT production in a circuit-localized mechanism. Glial serotonergic signaling is received by 5-HT_2A_ receptors on the astrocyte-like glia in a glia-to-glia communication mechanism (Figure 2D). In astrocytes, 5-HT_2A_ receptors act upstream of the production of secreted matrix metalloproteinase 1 (MMP1), which is responsible for the highly localized degradation of extracellular matrix (ECM; Miller and Broadie, 2024, 2025). Directed degradation permits the phagocytic glia to infiltrate the synaptic neuropil and prune targeted synapses (Figure 2D). The causal relationship between astrocyte glial 5-HT_2A_ receptor signaling, MMP function, and critical period experience-dependent synapse pruning was established by compensating for astrocytic 5-HT_2A_ receptor loss by the cell-targeted genetic expression of MMP-1 (Figure 2D; Miller and Broadie, 2024). Thus, glia serotonergic modulation plays an essential role in critical period synapse pruning via an experience-driven glia-to-glia subtype communication mechanism dependent on astrocyte 5-HT_2A_ receptors required for targeted synapse elimination.

## Serotonin signaling for glial mediated synaptic pruning

**Figure 3:**
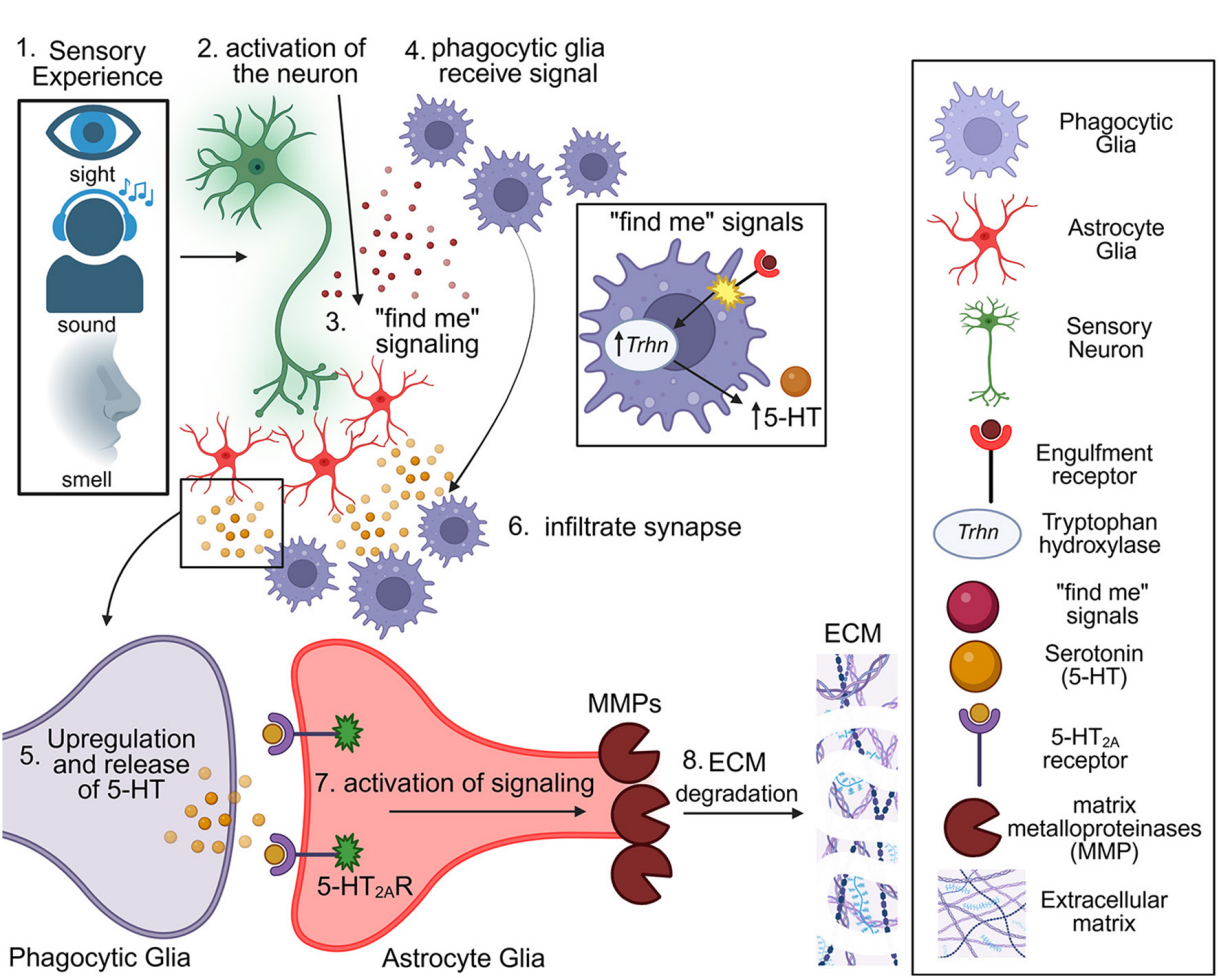
Glia-to-glia subtype serotonergic 5-HT_2A_ receptor signaling mediates synapse pruning. Different sensory modalities (e.g., sight, sound, smell) activate their sensory neurons **(1–2)**, triggering release of long-range “find-me” guidance signals **(3)** that recruit phagocytic glia **(4)**. In response to these targeting cues, phagocytic glia upregulate the 5-HT biosynthetic enzyme tryptophan hydroxylase (Trhn) to elevate 5-HT production **(5)**. For glial infiltration **(6)**, serotonin is released to signal astrocyte-like glia via 5-HT_2A_ receptors at as yet undefined cellular locations, initiating intracellular signaling to drive matrix metalloproteinase (MMP) production and release **(7)**. MMP-dependent degradation of the perineuronal extracellular matrix (ECM) removes the structural barrier surrounding synaptic neuropil **(8)**, enabling phagocytic glia to infiltrate, engulf and phagocytose targeted synapses. Note this model is derived from *Drosophila* studies. Right, cell types and molecular components include phagocytic glia, astrocyte glia, sensory neurons, engulfment receptors, Trhn, “find-me” signals, serotonin (5-HT), 5-HT_2A_ receptors, MMPs, and ECM. Together, this pathway provides a coordinated intercellular signaling mechanism in which sensory experience induces glial serotonin synthesis and 5-HT receptor signaling to grant glia phagocytes selective access to synapses marked for experience-dependent pruning.

Glia are the most abundant, yet still largely overlooked, cells of the nervous system (Figure 3) (Herculano-Houzel, 2009; von Bartheld et al., 2016). Glial cells are diverse in both morphology and function across the nervous system. In mammals, there are 5 main subtypes (astrocytes, microglia, ependymal, oligodendrocytes, and oligodendrocyte precursor cells) and 3 specialized regional glia (radial, Bergmann, and Müller glia; Wang et al., 2012; Vecino et al., 2016; Allen and Lyons, 2018; Ludwig and Das, 2023). Only two glial classes have known roles in serotonergic signaling or activity-dependent synaptic plasticity; astrocytes and microglia (Ota et al., 2013). Astrocytes, enormously diverse but named for “star-shaped” morphologies, are very closely associated with synapses and have key functions in synaptic modulation (Figure 3) (Chung et al., 2015; Lyon and Allen, 2022) Astrocytes are a core component of the tripartite synapse, together with a presynaptic bouton and postsynaptic dendrite, placing them as active partners in synaptic signaling and activity-dependent plasticity (Araque et al., 1999). Indeed, gliotransmission, including the Ca^2+^-dependent release of neurotransmitters, demonstrates an active astrocyte signaling function (Parpura et al., 1994; González-Arias et al., 2023). Microglia function as phagocytes in the brain immune response and, together with astrocytes, can prune synapses (Figure 3) (Faust et al., 2021; Lee et al., 2022; Chung et al., 2024). Glia actively engulf synapses in both the developing and mature brain, responding to neuron-to-glia targeting directional signals (Schafer et al., 2013; Nelson et al., 2024, 2025). This dynamic mechanism of glial pruning is required in juvenile neural circuit connectivity remodeling (Figure 3), with dysfunction in synaptic pruning highly implicated in several neurodevelopmental disorders (Hong et al., 2016b; Andoh and Koyama, 2021; Faust et al., 2021). It was recently discovered that such synaptic remodeling in mice involves glia-to-glia crosstalk between astrocytes and microglia via the Wnt signaling pathway (Faust et al., 2025). This signaling reduces astrocyte-synapse contact prior to microglia engulfment, demonstrating coordinated glia-to-glia intercellular communication (Figure 3), a mechanism similar to the serotonergic signaling recently discovered in *Drosophila*. Together, these mechanisms appear to be complementary to glia-to-glia communication, with intriguing similarity to mechanisms that involve coordinated functions across different glial classes.

**Figure 4:**
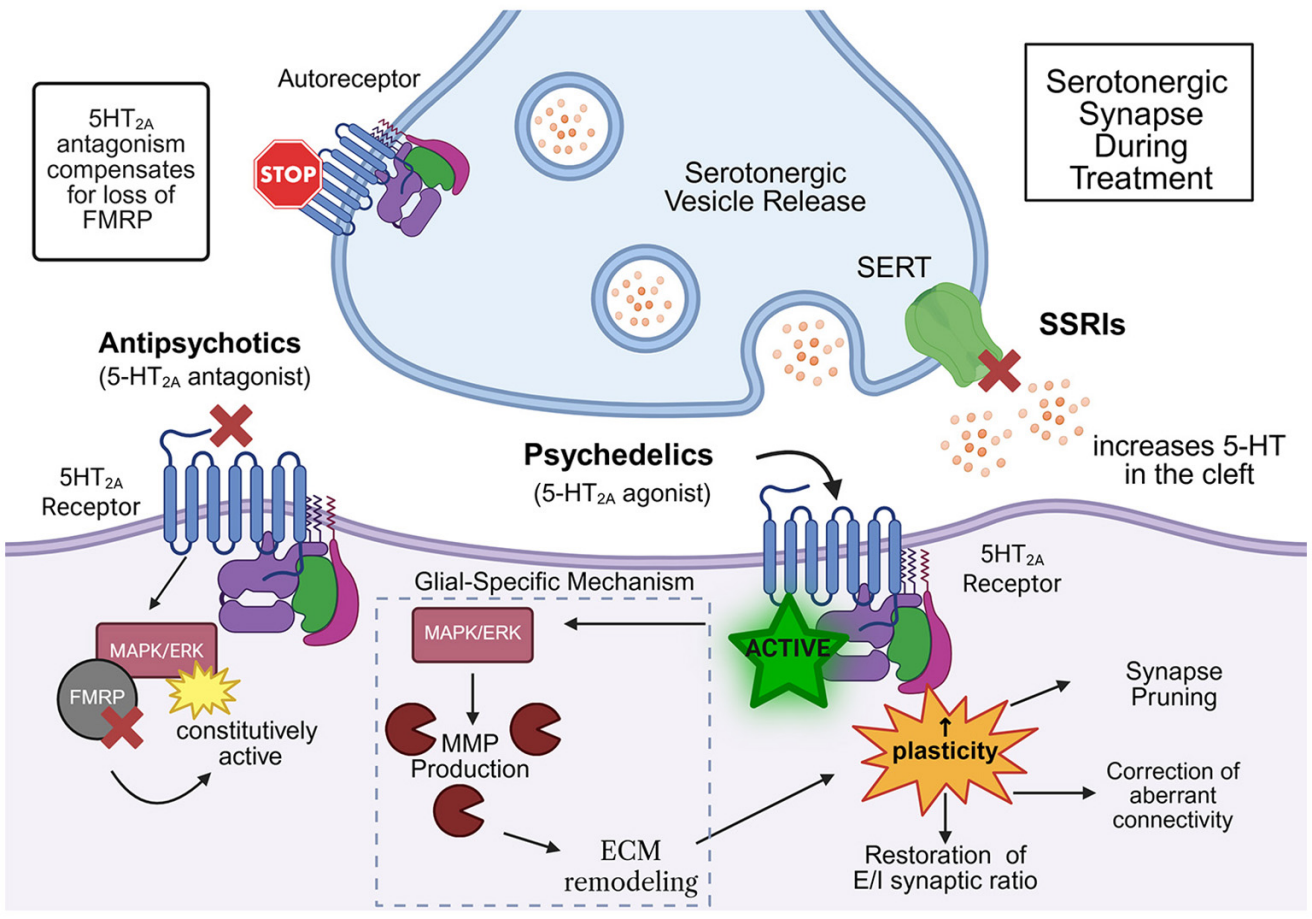
Pharmacological serotonergic modulation may re-open “critical period-like” plasticity. Schematic of serotonergic synapse function modified by treatment with common drug classes that target serotonin (5-HT) signaling. **(Left)** antipsychotics act as 5-HT_2A_ receptor antagonists, blocking aberrantly elevated signaling. In Fragile X syndrome (FXS), loss of FMRP leads to dysregulated MAPK/ERK signaling and persistent plasticity defects. 5-HT_2A_ antagonists compensates for the loss of FMRP and normalizes overactive pathways. **(Center)** psychedelics act as 5-HT_2A_ receptor agonists, activating MAPK/ERK signaling. This potential mechanism promotes matrix metalloproteinase (MMP) production and extracellular matrix (ECM) remodeling via glial function. This signaling cascade enables targeted synapse pruning, restores excitation/inhibition (E/I) synaptic balance, and corrects immature dendritic spine formation, together reinstating a state of heightened plasticity. **(Right)** selective serotonin reuptake inhibitors (SSRIs) inhibit SERT to increase 5-HT abundance, broadly potentiating serotonergic signaling. Elevated 5-HT levels can compensate for depressed 5-HT receptor expression. Collectively, these pharmacological strategies exploit different facets of 5-HT_2A_ receptor signaling, with emerging evidence suggesting that cell-type-specific glial 5-HT_2A_ receptor activation may produce a precision strategy to induce plasticity without the systemic consequences of global serotonin elevation.

*Drosophila* has multiple glial classes with strong function-based homology to mammalian glia (Freeman, 2015). There are 5 main classes of *Drosophila* glia (astrocyte-like glia, ensheathing glia, cortex glia, perineural glia, subperineural glia), and like mammals, 4 specialized regional glial classes (epifascicular, peripheral, satellite/cap, and midline glia; Awasaki et al., 2008). *Drosophila* glia are differentially named and classified based on their morphology and spatial distribution, however their functions tightly overlap with mouse glia (Figure 3) (Hartenstein, 2011). The two *Drosophila* glia classes mediating synaptic remodeling are the ensheathing glia (EG) and astrocyte-like glia (ALG; Nelson et al., 2024, 2025). Similar to mammalian astrocytes, astrocyte-like glia reside in tripartite synapses and mediate gliotransmission to shape synaptic function (Danjo et al., 2011; Macnamee et al., 2016). Ensheathing glia surround all the brain synaptic neuropils to infiltrate as activated phagocytes (Figure 3) (Doherty et al., 2009; Omoto et al., 2015), using the Draper engulfment receptor to phagocytose synapses both within injury and development contexts (MacDonald et al., 2006). In the *Drosophila* olfactory critical period, odorant experience activates the sensory neurons (Figure 3.1,2), which drives the release of “find me” signaling cues to direct phagocytic glia toward target synapses (Figure 3.3,4). *Drosophila* ensheathing glia, like mouse microglia, respond to “find me” signals that drive infiltration, migration, and directed engulfment of target synapses (Nelson et al., 2025). In response, ensheathing glia upregulate serotonin synthesis, which is then released as a signal to bind the 5-HT_2A_ receptors on astrocyte-like glia (Figure 3.5,6). Similar to mice, this glia-to-glia crosstalk is proposed to reduce the astrocyte-synapse contact prior to phagocytic glial engulfment (Faust et al., 2025). Astrocytic 5-HT_2A_ receptor signaling triggers MMP synthesis for degradation of the perineuronal ECM (Figure 3.7,8); Miller and Broadie, 2024), thus removing the physical barrier to phagocytic glia infiltration and enabling subsequent experience-dependent synapse pruning during the early-life critical period.

Glial pruning is mediated via many phagocytosis modes, including synaptomatrix destabilization, direct synapse engulfment, and fragmentation scavenging (MacDonald et al., 2006; Wilkinson and El Khoury, 2012; Crapser et al., 2021). Different serotonin receptors (5-HT_2_, 5-HT_4_, 5-HT_7_) bias downstream phagocytic receptor expression (complement receptors, scavenger receptors like MSR1/CD36), protease or chemotactic signal release, and actin dynamics (Liu et al., 2016; Zheng and Xu, 2025). For synaptic pruning, complement (C1q/C3/CR3) and phosphatidylserine engulfment pathway cross-talk is another determinant (Schafer et al., 2012; Scott-Hewitt et al., 2020). Serotonergic signaling can change ECM protease activity and perineuronal net (PNN) composition, thereby altering pruning constraints, with serotonin elevation (fluoxetine exposure) extending “critical period-like” plasticity by blocking the PNN deposition that normally delineates critical period closure (Mukhopadhyay et al., 2021). For experience-dependent glial pruning, the phagocytic glia must gain access to the target synapses, which is otherwise blocked by the ECM barrier (Figure 3). Therefore, ECM must be degraded or remodeled for accessibility, typically via secreted matrix metalloproteases (Nguyen et al., 2020; Crapser et al., 2021). During the *Drosophila* early-life olfactory critical period, secreted MMP-1 is both upregulated and required for experience-dependent pruning of sensory neuron synapses, with the knockdown of MMP-1 in astrocyte-like glia completely blocking this pruning (Figure 3). Moreover, the blocked pruning that results from the loss of astrocyte 5-HT_2A_ receptors can be entirely restored through astrocytic MMP-1 overexpression (Miller and Broadie, 2025). This finding provides strong evidence that the 5-HT_2A_ receptor function within astrocyte-like glia is to upregulate MMP-1 to enable ECM barrier degradation for remodeling (Figure 3). Taken together, the currently proposed mechanism is that critical period sensory experience elevates 5-HT release from phagocytic glia, which binds astrocyte-like glia 5-HT_2A_ receptors to drive MMP induction, which promotes localized ECM remodeling, thus enabling access of infiltrating phagocytic glia to prune target synapses in a temporally-restricted mechanism.

## Serotonergic signaling to re-open “critical period-like” plasticity

Serotonin (5-HT) is a key neuromodulator known to drive plasticity across development and throughout later life (Gaspar et al., 2003). As early as the 1950s, the idea emerged that many neuropsychiatric disorders may be caused by a deficit in serotonergic signaling, when Woolley (1958) hypothesized the link between serotonin dysregulation and the later emergence of behavior disabilities. Although framed exclusively from a neuronal perspective, recent studies suggest glial serotonergic signaling is also important. The connection to psychoactive drugs was first introduced with psychedelic drug trials (Osmond, 1957). When the Swiss chemist Albert Hofmann accidentally discovered lysergic acid diethylamide (LSD), new research was catapulted into the field of serotonergic signaling neuroplasticity (Figure 4; Nichols, 2016). The 1960s and 1970s recreational drug use led to “Schedule I” drug classifications, which halted most research. Organizations such as Multidisciplinary Association for Psychedelic Research Studies (MAPS) worked to re-legitimize research (Wark and Galliher, 2010; Kenny et al., 2023). A major finding was that psychedelics, including LSD and psylocibin, function as 5HT_2A_ receptor agonists (Figure 4), and these drugs appear to drive elevated plasticity (Grieco et al., 2022; Weiss et al., 2025). By the 1990s, mounting evidence emphasized the importance of serotonergic signaling (Bremshey et al., 2024), including the delayed and prolonged effectiveness of antidepressants, causing the formulation of the new “neuroplasticity hypothesis” (Benatti et al., 2024; Cui et al., 2024). This hypothesis proposes that major depressive disorder is caused by a decrease in neuroplasticity, and that serotonergic anti-depressants work by restoring this experience-dependent adaptability (Figure 4; Duman et al., 1997; Malberg et al., 2000). Subsequent studies showed that mouse hippocampal neurons plated with LSD exhibit strong 5-HT_2A_ receptor agonism that results in elevated *de novo* synaptogenesis (Ly et al., 2018). This extensive research history has led to the exciting proposal that serotonergic signaling acts as an essential gating mechanism in neuroplasticity mechanisms across timescales of aging (Figure 4). Although this hypothesis was proposed for neuronal serotonergic signaling, recent work suggests glia also play a central role in this mechanism.

The normally temporally-transient critical periods of heightened synaptic plasticity enable input experience to strengthen/weaken connections to optimize circuit function (Espinosa and Stryker, 2012). These synaptic changes can be very large-scale, reversible only during the critical period, with critical period closure permanently consolidated in the circuit (Golovin and Broadie, 2016; Nelson et al., 2024). Thus, when mechanisms underlying critical period opening, maintenance, or closure are dysfunctional, life-long circuit impairments result (Doll and Broadie, 2014; Nelson et al., 2025). Seemingly irreparable negative clinical outcomes are particularly linked to the normal permanent closure of critical period circuit remodeling capabilities (Irwin et al., 2000; Takesian et al., 2018; Larsen et al., 2023; Shang and Zhang, 2025). Pharmacological interventions with drugs targeting serotonergic signaling represent an attractive strategy to re-open “critical period-like” plasticity (Figure 4). This includes glial targets of such drugs. Serotonin is implicated as a vital participant in harnessing plasticity via selective serotonin reuptake inhibitor (SSRI) use. SSRIs bind to the central substrate-binding pocket (S1) of the SERT transporter, which blocks the reuptake of serotonin to increase extracellular 5-HT concentration in the synaptic cleft (Figure 4; Kristensen et al., 2011). Chronic SSRI use also reduces inhibitory tone through BDNF/TrkB-dependent changes in inhibitory interneurons (Moliner et al., 2023). Fragile X syndrome patients show significant cognitive and behavioral improvements with SSRI treatments (Kim et al., 2022; Ciranna and Costa, 2025). Chronic SSRI treatment is now strongly supported as a neuroplasticity-promoting intervention rather than a simple “chemical imbalance” fix, based on converging evidence from rodents and *Drosophila* (Figure 4). In mice and rats, prolonged fluoxetine treatment enhances adult hippocampal neurogenesis, accelerates maturation of newborn granule cells, and strengthens synaptic plasticity, providing a mechanistic substrate for durable antidepressant and cognitive effects (Wang et al., 2008). Fluoxetine also increases BDNF and BDNF-dependent synaptic proteins in hippocampus, prefrontal cortex, and other relevant brain regions, with SSRIs driving coordinated upregulation of BDNF/LTP-associated genetic programs (O'Leary et al., 2009; Hodes et al., 2010; Sachs and Caron, 2014).

Such plasticity effects are behaviorally relevant. SSRIs ameliorate cognitive deficits and stress- or diet-induced impairments in mice in a BDNF/CREB-dependent manner, and manipulations of CREB or BDNF signaling in serotonergic or hippocampal neurons blunt the efficacy of fluoxetine (Zuo et al., 2024). At the systems level, transcriptomic work demonstrates that chronic fluoxetine treatment induces a juvenile-like “dematuration” state in hippocampus and prefrontal cortex, reopening key forms of experience-dependent plasticity that align with the “network re-tuning” model of antidepressant action (Figure 4; Hagihara et al., 2019). Based on *Drosophila* studies, it appears glial serotonergic signaling may be part of this mechanism. *Drosophila* studies provide complementary circuit-level support directly linking 5-HT production and SERT manipulation to learning/memory (Neckameyer et al., 2007). Neuronal serotonin synthesis is controlled by tryptophan hydroxylase (Trhn), and mutant loss of 5-HT production impairs locomotor and olfactory behaviors, establishing the necessity of endogenous 5-HT signaling for normal sensorimotor and cognitive performance (Neckameyer et al., 2007). Behavioral and circuit studies show that serotonergic signaling in the *Drosophila* mushroom body and associated modulatory neurons mediates place memory, olfactory learning, and long-term memory, with 5-HT receptor RNAi in MB output neurons disrupting memory consolidation (Sitaraman et al., 2008). These neurons express the *Drosophila* serotonin transporter (dSERT), and recent genetic and transcriptomic studies using *dSERT* loss-of-function alleles, SSRI citalopram dSERT inhibition, and dSERT-targeted RNAi demonstrate perturbed sleep, feeding, and mushroom body Kenyon cell gene expression in ways that reproduce SSRI-induced mammalian circuit remodeling (Figure 4; Bonanno and Krantz, 2023). Moreover, blocking dSERT pharmacologically with fluoxetine, or otherwise elevating extracellular 5-HT, reshapes olfactory circuit odorant input gain control and sensory throughput, providing a striking *Drosophila* parallel to SSRI-induced reweighting of sensory and limbic circuits that underlies therapeutic network reorganization (Figure 4; Suzuki et al., 2020; Gajardo et al., 2023).

Taken together, both mouse and *Drosophila* findings argue that by blocking SERT and enhancing serotonin tone, SSRIs engage conserved 5-HT-dependent mechanisms of transcriptomic and synaptic plasticity for circuit reconfiguration that supports clinical antidepressant effects, as well as learning and memory (Figure 4). Specific 5-HT receptors are connected to brain circuit connectivity remodeling. In the *Drosophila* olfactory critical period, adult conditional 5-HT_2A_ receptor overexpression in astrocyte-like glia re-opens “critical period-like” circuit remodeling based on a structural connectivity level, based on experience-dependent synapse pruning (Miller and Broadie, 2025). 5-HT_2A_Rs were genetically manipulated in a temporally-controlled manner, with overexpression only in adulthood (Figure 4). Similarly, 5-HT_2A_ receptor agonists exert effects by activating Gq/11-coupled 5-HT_2A_ receptors, which in turn stimulates PLC, leading to IP3-mediated intracellular Ca^2+^ release and PKC activation, a signaling cascade that powerfully modulates cortical excitability and plasticity (Wallach et al., 2023). Current efforts focus on the use of 5-HT_2A_ receptor agonists, such as LSD and MDMA, provided globally to upregulate plasticity in adults (Figure 4; Inserra et al., 2021). The undervalued role of glia-to-glia signaling in critical period remodeling may open the door to more targeted 5-HT_2A_ receptor agonist therapeutic interventions (Faust et al., 2025; Miller and Broadie, 2025). In plated mouse hippocampal neurons, *de novo* synaptogenesis follows exposure to 5-HT_2A_ receptor agonists including LSD, MDMA, and ketamine (Figure 4; Ly et al., 2018; Saeger and Olson, 2022). In clinical studies, there is a significant decrease in PTSD-related nightmares in patients with tightly controlled microdosed treatments of MDMA (Mitchell et al., 2021). Such studies propose that 5-HT_2A_ receptor agonist activity during guided therapy sessions may enable synaptogenesis and rewiring within affected circuits (Figure 4). However, 5-HT_2A_ receptor agonist treatments could also result in non-specific synapse formation and pruning changes that could potentially further worsen, or even initiate, brain circuit disorders (Grieco et al., 2022; Weiss et al., 2025). Importantly, note again that serotonergic modulation mediates differential mechanisms in specific cell types, therefore treatments need to be refined to reflect the same cellular specificity when treating mis-wired circuits (Aghajanian and Marek, 1997; Agahari and Stricker, 2025).

At the mechanistic level, recent work shows that classic psychedelics such as psilocybin, LSD, and DMT act as 5-HT_2A_ receptor agonists to function as “psychoplastogens,” promoting rapid and robust neuritogenesis, dendritic spine growth, and synaptogenesis in cortical neurons (Ly et al., 2018; Jiang et al., 2026; Figure 4). 5-HT_2A_ receptor signaling via membrane-permeant psychedelics selectively activates the intracellular receptor pool to drive cortical structural and functional neuroplasticity, identifying this 5-HT_2A_ receptor pool as a potential key therapeutic target (Figure 4; Vargas et al., 2023). 5-HT_2A_ receptor agonist long-lasting therapeutic effects across mood and substance-use disorders are best explained as sustained enhancements of synaptic plasticity (Grieco et al., 2022). In mice, multiple psychedelics re-open a closed social-reward learning critical period, with re-opening closely tracking the 5-HT_2A_ receptor-dependent psychedelic time course for each drug, thus linking 5-HT_2A_ agonism to “critical period-like” plasticity (Figure 4; Nardou et al., 2023). Clinically, psilocybin (a selective 5-HT_2A_ receptor agonist at therapeutic doses) has rapid and sustained antidepressant effects, with a phase 2 double-blind trial in treatment-resistant depression finding a single 25-mg dose produces significantly improvements, with benefits persisting in a subset of patients (Goodwin et al., 2022). In major depressive disorder, a randomized, placebo-controlled trial similarly showed that one 25-mg psilocybin dose with psychological support produced a rapid and clinically meaningful reduction in depressive symptoms and functional disability, compared with placebo treatment (Figure 4), without serious adverse events (Raison et al., 2023). Collectively, these mechanistic, preclinical, and clinical data strongly support the therapeutic promise of 5-HT_2A_ receptor agonists for brain circuit remodeling clinical applications. By engaging cell-specific and pathway-biased 5-HT_2A_ receptor signaling to drive durable synaptic and network remodeling, these drugs potentially can re-open windows of plasticity. Taken together, serotonergic drugs offer a qualitatively different, plasticity-centered route to treating circuit-based disorders compared to traditional treatments.

Other circuit disorders such as schizophrenia, characterized by uncontrolled synaptic pruning in early adulthood, are treatable with 5-HT_2A_-targeted antipsychotics (Duan et al., 2024; Kargbo, 2024). 5-HT_2_A receptor antagonists bind an orthosteric site to stabilizes the Gq/11-coupled GPCR inactive conformation (Figure 4; Casey et al., 2022). Fragile X syndrome shows some cognitive and behavioral improvements with 5-HT_2A_ receptor antagonists (Kim et al., 2022; Ciranna and Costa, 2025). Notably, interventions are more successful when administered during critical periods, and can last into adulthood without further treatment (Greiss Hess et al., 2016). The efficacy of both agonists and antagonists seems contradictory, however miswiring results from disruptions in 5-HT homeostasis (Ocana-Santero et al., 2025), with serotonin signaling influenced by 5-HT reception and detection (Colwell et al., 2024). One potential hypothesis for this contradiction is that hyperexcitability of the sensory system from FXS aberrant wiring can be quieted either through the blocking of receptors, with prolonged receptor binding also resulting in endocytosis and removal of receptors form the membrane (Schaefer et al., 2015; Liu et al., 2021). Another hypothesis is different levels of circuitry mechanisms, with the antagonist may acting to control hypersensitivity and the antagonist enabling pruning to decrease hyperexcitability from supernumerary synapses (Campa et al., 2015; Deo and Redpath, 2022). A major concern is that serotonergic drugs have known harmful side effects, including cardiovascular risks, metabolic dysfunction, and movement disorders (i.e., tardive dyskinesia; Yekehtaz et al., 2013; Cornett et al., 2017; Mortimer et al., 2023). While such interventions remain promising, they lack the level of specificity required to provide efficient treatment for optimal outcomes. The new concept that glia are an essential cell type for serotonergic signaling in synaptic plasticity provides a possible avenue for therapeutic specificity (Figure 4). Utilizing tools to target glial 5-HT_2A_ receptors could limit side effects (Gleichman et al., 2023; Aoki et al., 2025), with the goal of generating safe treatments for circuit-based disorders in children and young adults. Harnessing the specificity in glia-to-glia serotonergic signaling suggests a way for therapeutics to treat circuit-based neurodevelopmental disorders (Figure 4). Cell-targeted serotonergic signaling interventions as a means to re-open “critical period-like” synaptic connectivity remodeling may provide a means for a new therapeutics approach to better treat debilitating circuit dysfunction neurological disorders.

## Discussion

Decades of work have shown that serotonin modulates neural circuit assembly and remodeling, however recent discoveries produce a seismic shift: serotonin does not act solely within neurons, but also functions in glia-to-glia signaling to enable critical period experience-dependent synapse pruning (Miller and Broadie, 2024; Gu et al., 2025). Specifically, in *Drosophila*, the discovery that glial serotonin production, glial 5-HT_2A_ receptor activation, and glial MMP-dependent extracellular matrix remodeling form an intercellular signaling pathway to transduce sensory experience into a temporally-transient critical period of access for glial infiltration phagocytosis. This work generates a directly testable mechanistic framework with clear translational implications: glia-directed serotonergic signaling may represent a conserved gating switch determining whether brain circuits are permissive or resistant to connectivity remodeling. The future challenge, and opportunity, lies in determining how this mechanism is experience-triggered, temporally-regulated, and potentially harnessed across species. The demonstration that tryptophan hydroxylase-dependent serotonin biosynthesis in phagocytic glia is essential for pruning suggests that glia act as sensors of sensory experience (Miller and Broadie, 2024; Nelson et al., 2024). However, the nature of the upstream experiential signal that induces glial serotonergic activation remains unknown. The hypothesis that insulin-like growth factors (IGF) released from activated neurons may serve as “find-me” cues is compelling, given their dual roles in glial recruitment and synaptic remodeling across species (Vita et al., 2021; Nelson and Broadie, 2025). Defining whether insulin receptor (InR) activation increases Trhn function directly, and determining which intracellular programs can couple IGF signaling to serotonin production, will be essential for understanding how sensory experience recruits the glial pruning machinery. This work also carries strong translational potential: if in fact IGF and serotonergic signaling are mechanistically linked during neural circuit remodeling, glial metabolic dysfunction could represent an under-recognized cause of neurodevelopmental pathology.

Recent findings in *Drosophila* elevate the glial 5-HT_2A_ receptor from a peripheral modifier of neuromodulation to a mechanistic control gate for developmental synaptic refinement (Miller and Broadie, 2024). However, the intracellular signaling cascade connecting 5-HT_2A_ receptor activation to pruning remains as yet undefined. Evidence from psychedelic studies and injury-induced plasticity suggests a role for MAPK/ERK signaling downstream of 5-HT_2A_ receptors (López-Giménez and González-Maeso, 2018; Baumann et al., 2024), but this has not been tested during experience-dependent synapse remodeling. Determining whether MAPK/ERK activation is necessary and sufficient for pruning—and whether it modulates protease secretion, cytoskeletal dynamics, or phagocytic machinery—should provide key insights into how glial activation states reshape circuitry (Chen et al., 2021; Baumann et al., 2024). Resolving the glial signaling axis should also enable us to distinguish plasticity-promoting serotonergic mechanisms from those responsible for general hallucinogenic or mood-altering effects, a prerequisite for targeted therapeutics (Grieco et al., 2022). The discovery that astrocyte-derived MMP function is necessary for critical period experience-dependent synaptic pruning positions ECM remodeling not as a downstream epiphenomenon, but rather as a rate-limiting step for experience-driven brain circuit remodeling (Miller and Broadie, 2025). The ability of MMP expression in guard glia to restore pruning by 5-HT_2A_ receptor null phagocytic glia strongly suggests a direct pathway of serotonergic signaling to activate ECM-degrading proteases and enable access to targeted synapses (Miller and Broadie, 2025). The field would need to determine the composition of this postulated ECM infiltration barrier and the exact nature of critical period glial remodeling (Tewari et al., 2022). Future work should determine if the sequence of 5-HT_2A_R activation → MAPK/ERK → MMP release → ECM degradation → phagocyte infiltration represents a conserved mechanism across species. This work suggest that neuronal activity selects synapses for targeted removal, but that glial access to those synapses is constrained, implying that ECM remodeling serves as the physical gate that determines when and where pruning can occur.

At the behavioral output level, serotonergic manipulations enhancing signaling prevent strong learning/memory deficits in the *Drosophila* Fragile X syndrome (FXS) disease model (Du et al., 2026). Upregulation of serotonin synthesis by *Trhn* overexpression or extracellular 5-HT elevation by SERT knockdown restores normal learning and memory abilities in this model. Importantly, glial function can act as the source of this serotonergic signaling. Future studies should work to determine whether this is a critical period role, setting the stage for later learning/memory abilities (Ogelman et al., 2024), or a direct plasticity role, providing serotonin at sufficient levels to enable the capacity for learning acquisition and memory consolidation (Higa et al., 2022; de Cates et al., 2023). Clinically, SSRIs inhibit SERT to raise 5-HT tone, and serotonergic modulation has been discussed in FXS clinical and preclinical contexts as a means to alleviate symptoms (Ciranna and Costa, 2025), with low-dose sertraline treatments in young FXS children providing benefit (Greiss Hess et al., 2016). In the *Drosophila* FXS model, future work is needed to test SSRI pharmacological interventions in both critical period and acute adult experiments. The apparent underlying cause of impaired serotonergic signaling is loss of 5-HT_2A_ receptors in the mushroom body learning/memory circuit of the *Drosophila* FXS model, with 5-HT_2A_ receptor overexpression preventing FXS learning/memory deficits (Du et al., 2026). There needs to be cell-targeted studies to dissect circuit-localized serotonergic signaling in this model. In mammals, 5-HT_2A_ receptors act as a plasticity switch, with receptor activation central to psychedelic-linked cortical plasticity (Vargas et al., 2023) In *Drosophila* and mouse FXS models, it is important to test 5-HT_2A_R agonists in trials of both critical period and acute adult interventions. Conversely, a 5-HT_2_ family antagonist (pirenperone) prevents behavioral deficits including impaired spatial memory in the mouse FXS model (Kim et al., 2022), again suggesting that serotonergic manipulations apparently in opposite directions may be effective in FXS disease models and possibly in future treatments of FXS patients (Pan et al., 2004; Dear et al., 2017).

In another behavioral setting, serotonin signaling has long been closely related to sleep behavior. 5-HT is not only a precursor of melatonin, which is a key regulator of sleep onset, but can also directly regulate sleep across species, including *Drosophila* (Borbély et al., 1981; Ursin et al., 1989). Future work should test the relationship between glial serotonergic signaling and heightened juvenile sleep in *Drosophila*. In mammals, 5-HT generally promotes wakefulness and inhibits sleep, yet the different 5-HT receptors have opposing effects on sleep (Monti, 2011). In mice, 5-HT_2A_ receptor knockout significantly increases wakefulness and selectively reduces slow-wave sleep, and 5-HT_2A/C_ blockade reduces overall sleep (Popa et al., 2005). Conversely, 5-HT signaling is also under the regulation of sleep. Future studies should test if sleep regulates serotonergic signaling and 5-HT receptor classes in the *Drosophila* model. In humans, sleep deprivation elevates 5-HT release (Davies et al., 2014), and mouse sleep deprivation upregulates 5HT_2A_ receptors (Zhao et al., 2022). Importantly, sleep is a well-known regulator of synaptic plasticity. Moving forward, studies could test whether sleep regulates experience-dependent synaptic pruning in the *Drosophila* genetic model. In numerous animal models, slow wave sleep is closely linked to a decrease in synapse size and numbers, with wakefulness increasing synapse number and strength (De Vivo et al., 2017; Liu et al., 2024). The homeostasis hypothesis posits that sleep regulates synapse connectivity (Tononi and Cirelli, 2006), with sleep deprivation in mouse critical periods blocking synapse pruning (Li et al., 2017). Recent studies find sleep deprivation dysregulates glial CX3CR1 signaling to inhibit synapse elimination (Vanrobaeys et al., 2023; Wang et al., 2023; Ni et al., 2025). Expansive future studies could test whether sleep regulates *Drosophila* glial synaptic pruning, and particularly if it does so through control of the glia-to-glia class serotonergic signaling at the level of 5HT_2A_ receptor-mediated MMP induction for glial pruning (Miller and Broadie, 2024; Gu et al., 2025) or at the level of direct engulfment glial phagocytosis (Vita et al., 2021; Miller and Broadie, 2024; Nelson et al., 2024; Nelson and Broadie, 2025).

Perhaps the most provocative discovery for the field is the finding that in *Drosophila*, adult conditional 5-HT_2A_ receptor expression in astrocyte-like glia is sufficient to reinstate “critical period-like” plasticity at maturity (Miller and Broadie, 2025). This supports the emerging view that mature circuits retain latent plasticity capacities that can be triggered by modulating glial state, rather than by altering neuron function alone (Ribot et al., 2021; Marciante et al., 2024). Glia-to-glia communication could be the gating variable enabling circuit remodeling (Faust et al., 2025). Determining whether adult plasticity utilizes the same serotonergic pathway MAPK/ERK → MMP axis as juvenile experience-dependent synapse pruning (Baumann et al., 2024), or whether distinct adult-specific pathways are engaged (Akol et al., 2022), is key for understanding both basic mechanism and clinical application. The ability to re-open connectivity remodeling via cell-selective 5-HT_2A_ activation suggests a route to therapeutic benefits of psychedelic-induced plasticity without widespread consequences (Cunningham et al., 2023; Vargas et al., 2023). Many neurological disorders, including FXS, ASD, and schizophrenia, are characterized by deficits in juvenile synapse pruning (Sellgren et al., 2019; Song and Broadie, 2023; Wu et al., 2024). Future work should test whether glial 5-HT_2A_R activation can correct pruning during early life or reinstate remodeling at maturity across brain circuits. Cell-type-specific agonists or gene therapy-based strategies aimed at astrocytic serotonergic signaling could represent a new class of interventions enabling this plasticity (González-Arias et al., 2023; Vargas et al., 2023; Miller and Broadie, 2024), avoiding the broad consequences of psychedelic pharmacology (Lewis et al., 2023; Sharp and Ippolito, 2025). Collectively, recent work suggests sensory experience triggers glial serotonergic signaling, which remodels the ECM to permit access to synapses marked for experience-dependent elimination. This conceptual shift has broad implications: pruning may not be limited by neuronal competence but rather glial infiltration. As the field continues to dissect mechanistic and translational consequences of this idea, glial-directed 5-HT modulation may emerge as a powerful lever to restore otherwise transient critical period capabilities.

## References

[B1] Agahari F. A. Stricker C. (2021). Serotonergic modulation of spontaneous and evoked transmitter release in layer II pyramidal cells of rat somatosensory cortex. Cereb. Cortex 31, 1182–1200. doi: 10.1093/cercor/bhaa285 33063109

[B2] Agahari F. A. Stricker C. (2025). Modulation by serotonin reveals preferred recurrent excitatory connectivity in layer II of rat neocortex. Cereb. Cortex 35:bhaf008. doi: 10.1093/cercor/bhaf008 39937460

[B3] Aghajanian G. K. Marek G. J. (1997). Serotonin induces excitatory postsynaptic potentials in apical dendrites of neocortical pyramidal cells. Neuropharmacology 36, 589–599. doi: 10.1016/S0028-3908(97)00051-8 9225284

[B4] Akol I. Kalogeraki E. Pielecka-Fortuna J. Fricke M. Löwel S. (2022). MMP2 and MMP9 activity is crucial for adult visual cortex plasticity in healthy and stroke-affected mice. J. Neurosci. 42, 16–32. doi: 10.1523/JNEUROSCI.0902-21.2021 34764155 PMC8741160

[B5] Albert P. R. Vahid-Ansari F. (2019). The 5-HT1A receptor: signaling to behavior. Biochimie 161, 34–45. doi: 10.1016/j.biochi.2018.10.015 31079617

[B6] Albertini G. D'Andrea I. Druart M. Béchade C. Nieves-Rivera N. Etienne F. . (2023). Serotonin sensing by microglia conditions the proper development of neuronal circuits and of social and adaptive skills. Mol. Psychiatry 28, 2328–2342. doi: 10.1038/s41380-023-02048-5 37217677

[B7] Allen N. J. Lyons D. A. (2018). Glia as architects of central nervous system formation and function. Science 362:181. doi: 10.1126/science.aat0473 30309945 PMC6292669

[B8] Andoh M. Koyama R. (2021). Microglia regulate synaptic development and plasticity. Dev. Neurobiol. 81, 568–590. doi: 10.1002/dneu.22814 33583110 PMC8451802

[B9] Ansorge M. S. Zhou M. Lira A. Hen R. Gingrich J. A. (2004). Early-life blockade of the 5-HT transporter alters emotional behavior in adult mice. Science 306, 879–881. doi: 10.1126/science.1101678 15514160

[B10] Antonini A. Stryker M. P. (1993). Rapid remodeling of axonal arbors in the visual cortex. Science 260, 1819–1821. doi: 10.1126/science.8511592 8511592

[B11] Aoki R. Konno A. Hosoi N. Kawabata H. Hirai H. (2025). AAV vectors for specific and efficient gene expression in microglia. Cell Rep. Methods 5:101116. doi: 10.1016/j.crmeth.2025.101116 40744011 PMC12461629

[B12] Araque A. Parpura V. Sanzgiri R. P. Haydon P. G. (1999). Tripartite synapses: Glia, the unacknowledged partner. Trends Neurosci. 22, 208–215. doi: 10.1016/S0166-2236(98)01349-6 10322493

[B13] Astill Wright L. Horstmann L. Holmes E. A. Bisson J. I. (2021). Consolidation/reconsolidation therapies for the prevention and treatment of PTSD and re-experiencing: a systematic review and meta-analysis. Transl. Psychiatry 11:453. doi: 10.1038/s41398-021-01570-w 34480016 PMC8417130

[B14] Awasaki T. Lai S. L. Ito K. Lee T. (2008). Organization and postembryonic development of glial cells in the adult central brain of *Drosophila*. J. Neurosci. 28, 13742–13753. doi: 10.1523/JNEUROSCI.4844-08.2008 19091965 PMC6671902

[B15] Azmitia E. C. (2001). Modern views on an ancient chemical: serotonin effects on cell proliferation, maturation, and apoptosis. Brain Res. Bull. 56, 413–424. doi: 10.1016/S0361-9230(01)00614-1 11750787

[B16] Bajar B. T. Phi N. T. Randhawa H. Akin O. (2022). Developmental neural activity requires neuron-astrocyte interactions. Dev. Neurobiol. 82:235. doi: 10.1002/dneu.22870 35225404 PMC9018619

[B17] Baumann N. S. Sears J. C. Broadie K. (2024). Experience-dependent MAPK/ERK signaling in glia regulates critical period remodeling of synaptic glomeruli. Cell. Signal. 120:11124. doi: 10.1016/j.cellsig.2024.111224 38740233 PMC11459659

[B18] Belmer A. Quentin E. Diaz S. L. Guiard B. P. Fernandez S. P. Doly S. . (2018). Positive regulation of raphe serotonin neurons by serotonin 2B receptors. Neuropsychopharmacology 43:1623. doi: 10.1038/s41386-018-0013-0 29453444 PMC5983540

[B19] Benatti B. M. Adiletta A. Sgadò P. Malgaroli A. Ferro M. Lamanna J. (2024). Epigenetic modifications and neuroplasticity in the pathogenesis of depression: a focus on early life stress. Behav. Sci. 14:882. doi: 10.3390/bs14100882 39457754 PMC11504006

[B20] Berger M. Gray J. A. Roth B. L. (2009). The expanded biology of serotonin. Annu. Rev. Med. 60, 355–366. doi: 10.1146/annurev.med.60.042307.110802 19630576 PMC5864293

[B21] Bonanno S. L. Krantz D. E. (2023). Transcriptional changes in specific subsets of *Drosophila* neurons following inhibition of the serotonin transporter. Transl. Psychiatry 13:226. doi: 10.1038/s41398-023-02521-3 37355701 PMC10290657

[B22] Bonnin A. Goeden N. Chen K. Wilson M. L. King J. Shih J. C. . (2011). A transient placental source of serotonin for the fetal forebrain. Nature 472, 347–350. doi: 10.1038/nature09972 21512572 PMC3084180

[B23] Bonnin A. Levitt P. (2011). Fetal, maternal, and placental sources of serotonin and new implications for developmental programming of the brain. Neuroscience 197, 1–7. doi: 10.1016/j.neuroscience.2011.10.005 22001683 PMC3225275

[B24] Borbély A. A. Neuhaus H. U. Tobler I. (1981). Effect of p-chlorophenylalanine and tryptophan on sleep, EEG and motor activity in the rat. Behav. Brain Res. 2, 1–22. doi: 10.1016/0166-4328(81)90035-8 6452888

[B25] Bourgeois J. P. Goldman-Rakic P. S. Rakic P. (1994). Synaptogenesis in the prefrontal cortex of rhesus monkeys. Cereb. Cortex 4, 78–96. doi: 10.1093/cercor/4.1.78 8180493

[B26] Bremshey S. Groß J. Renken K. Masseck O. A. (2024). The role of serotonin in depression-a historical roundup and future directions. J. Neurochem. 168, 1751–1779. doi: 10.1111/jnc.16097 38477031

[B27] Briner A. De Roo M. Dayer A. Muller D. Kiss J. Z. Vutskits L. (2010). Bilateral whisker trimming during early postnatal life impairs dendritic spine development in the mouse somatosensory barrel cortex. J. Comp. Neurol. 518, 1711–1723. doi: 10.1002/cne.22297 20235164

[B28] Brummelte S. Mc Glanaghy E. Bonnin A. Oberlander T. F. (2016). Developmental changes in serotonin signaling: implications for early brain function, behavior and adaptation. Neuroscience 342:212. doi: 10.1016/j.neuroscience.2016.02.037 26905950 PMC5310545

[B29] Cameron L. P. Benetatos J. Lewis V. Bonniwell E. M. Jaster A. M. Moliner R. . (2023). Beyond the 5-HT2A receptor: classic and nonclassic targets in psychedelic drug action. J. Neurosci. 43, 7472–7482. doi: 10.1523/JNEUROSCI.1384-23.2023 37940583 PMC10634557

[B30] Campa V. M. Capilla A. Varela M. J. De La Rocha A. M. A. Fernandez-Troyano J. C. Belén Barreiro R. . (2015). Endocytosis as a biological response in receptor pharmacology: evaluation by fluorescence microscopy. PLoS ONE 10:e0122604. doi: 10.1371/journal.pone.0122604 25849355 PMC4388511

[B31] Carreira-Rosario A. York R. A. Choi M. Doe C. Q. Clandinin T. R. (2021). Mechanosensory input during circuit formation shapes *Drosophila* motor behavior through patterned spontaneous network activity. Curr. Biol. 31, 5341–5349.e4. doi: 10.1016/j.cub.2021.08.022 34478644 PMC8665011

[B32] Carvajal-Oliveros A. Campusano J. M. (2021). Studying the contribution of serotonin to neurodevelopmental disorders. Can this fly? Front. Behav. Neurosci. 14:601449. doi: 10.3389/fnbeh.2020.601449 33510625 PMC7835640

[B33] Casey A. B. Cui M. Booth R. G. Canal C. E. (2022). “Selective” serotonin 5-HT2A receptor antagonists. Biochem. Pharmacol. 200:115028. doi: 10.1016/j.bcp.2022.115028 35381208 PMC9252399

[B34] Cavaccini A. Gritti M. Giorgi A. Gozzi A. Pasqualetti M. Correspondence R. T. (2018). Serotonergic signaling controls input-specific synaptic plasticity at striatal circuits. Neuron 98, 801–816. doi: 10.1016/j.neuron.2018.04.008 29706583

[B35] Chaumont-Dubel S. Dupuy V. Bockaert J. Bécamel C. Marin P. (2020). The 5-HT6 receptor interactome: new insight in receptor signaling and its impact on brain physiology and pathologies. Neuropharmacology 172:107839. doi: 10.1016/j.neuropharm.2019.107839 31682856

[B36] Chen M. J. Ramesha S. Weinstock L. D. Gao T. Ping L. Xiao H. . (2021). Extracellular signal-regulated kinase regulates microglial immune responses in Alzheimer's disease. J. Neurosci. Res. 99, 1704–1721. doi: 10.1002/jnr.24829 33729626 PMC8919593

[B37] Chen X. Kim Y. Kawaguchi D. (2025). Development of the rodent prefrontal cortex: circuit formation, plasticity, and impacts of early life stress. Front. Neural Circuits 19:1568610. doi: 10.3389/fncir.2025.1568610 40206866 PMC11979153

[B38] Christopherson K. S. Ullian E. M. Stokes C. C. A. Mullowney C. E. Hell J. W. Agah A. . (2005). Thrombospondins are astrocyte-secreted proteins that promote CNS synaptogenesis. Cell 120, 421–433. doi: 10.1016/j.cell.2004.12.020 15707899

[B39] Chung W. S. Allen N. J. Eroglu C. (2015). Astrocytes control synapse formation, function, and elimination. Cold Spring Harb. Perspect. Biol. 7:a020370. doi: 10.1101/cshperspect.a020370 25663667 PMC4527946

[B40] Chung W. S. Baldwin K. T. Allen N. J. (2024). Astrocyte regulation of synapse formation, maturation, and elimination. Cold Spring Harb. Perspect. Biol. 16:a041352. doi: 10.1101/cshperspect.a041352 38346858 PMC11293538

[B41] Ciranna L. Costa L. (2025). Therapeutic effects of pharmacological modulation of serotonin brain system in human patients and animal models of Fragile X syndrome. Int. J. Mol. Sci. 26:2495. doi: 10.3390/ijms26062495 40141138 PMC11941774

[B42] Colas J. F. Launay J. M. Maroteaux L. (1999). Maternal and zygotic control of serotonin biosynthesis are both necessary for *Drosophila* germband extension. Mech. Dev. 87, 67–76. doi: 10.1016/S0925-4773(99)00140-9 10495272

[B43] Colwell M. J. Tagomori H. Shang F. Cheng H. I. Wigg C. E. Browning M. . (2024). Direct serotonin release in humans shapes aversive learning and inhibition. Nat. Commun. 15:50394. doi: 10.1038/s41467-024-50394-x 39122687 PMC11315928

[B44] Cornett E. M. Novitch M. Kaye A. D. Kata V. Kaye A. M. (2017). Medication-induced tardive dyskinesia: a review and update. Ochsner J. 17:162. 28638290 PMC5472076

[B45] Coulson B. Hunter I. Doran S. Parkin J. Landgraf M. Baines R. A. (2022). Critical periods in *Drosophila* neural network development: importance to network tuning and therapeutic potential. Front. Physiol. 13:1073307. doi: 10.3389/fphys.2022.1073307 36531164 PMC9757492

[B46] Crapser J. D. Arreola M. A. Tsourmas K. I. Green K. N. (2021). Microglia as hackers of the matrix: sculpting synapses and the extracellular space. Cell. Mol. Immunol. 18, 2472–2488. doi: 10.1038/s41423-021-00751-3 34413489 PMC8546068

[B47] Cui L. Li S. Wang S. Wu X. Liu Y. Yu W. . (2024). Major depressive disorder: hypothesis, mechanism, prevention and treatment. Signal Transduct. Target. Ther. 9:194. doi: 10.1038/s41392-024-01738-y 38331979 PMC10853571

[B48] Cunningham M. J. Bock H. A. Serrano I. C. Bechand B. Vidyadhara D. J. Bonniwell E. M. . (2023). Pharmacological mechanism of the non-hallucinogenic 5-HT2A agonist ariadne and analogs. ACS Chem. Neurosci. 14, 119–135. doi: 10.1021/acschemneuro.2c00597 36521179 PMC10147382

[B49] Dai Y. Dudek N. L. Li Q. Muma N. A. (2010). Phospholipase C, Ca2+, and calmodulin signaling are required for 5-HT2A receptor-mediated transamidation of Rac1 by transglutaminase. Psychopharmacology 213:403. doi: 10.1007/s00213-010-1984-7 20717650 PMC3033764

[B50] Danjo R. Kawasaki F. Ordway R. W. (2011). A tripartite synapse model in *Drosophila*. PLoS ONE 6:e17131. doi: 10.1371/journal.pone.0017131 21359186 PMC3040228

[B51] Daubert E. A. Condron B. G. (2010). Serotonin: a regulator of neuronal morphology and circuitry. Trends Neurosci. 33, 424–434. doi: 10.1016/j.tins.2010.05.005 20561690 PMC2929308

[B52] Davies S. K. Ang J. E. Revell V. L. Holmes B. Mann A. Robertson F. P. . (2014). Effect of sleep deprivation on the human metabolome. Proc. Natl. Acad. Sci. U.S.A. 111, 10761–10766. doi: 10.1073/pnas.1402663111 25002497 PMC4115565

[B53] de Cates A. N. Martens M. A. G. Wright L. C. Gibson D. Spitz G. Gould van Praag C. D. . (2023). 5-HT4 receptor agonist effects on functional connectivity in the human brain: implications for procognitive action. Biol. Psychiatry Cogn. Neurosci. Neuroimaging 8, 1124–1134. doi: 10.1016/j.bpsc.2023.03.014 37098409 PMC10914664

[B54] De Vivo L. Bellesi M. Marshall W. Bushong E. A. Ellisman M. H. Tononi G. . (2017). Ultrastructural evidence for synaptic scaling across the wake/sleep cycle. Science 355:507. doi: 10.1126/science.aah5982 28154076 PMC5313037

[B55] Dear M. L. Shilts J. Broadie K. (2017). Neuronal activity drives FMRP- and HSPG-dependent matrix metalloproteinase function required for rapid synaptogenesis. Sci. Signal. 10:aan3181. doi: 10.1126/scisignal.aan3181 29114039 PMC5743058

[B56] Deo N. Redpath G. (2022). Serotonin receptor and transporter endocytosis is an important factor in the cellular basis of depression and anxiety. Front. Cell. Neurosci. 15:804592. doi: 10.3389/fncel.2021.804592 35280519 PMC8912961

[B57] Doherty J. Logan M. A. Taşdemir Ö. E. Freeman M. R. (2009). Ensheathing glia function as phagocytes in the adult *Drosophila* brain. J. Neurosci. 29:4768. doi: 10.1523/JNEUROSCI.5951-08.2009 19369546 PMC2674269

[B58] Doll C. A. Broadie K. (2014). Impaired activity-dependent neural circuit assembly and refinement in autism spectrum disorder genetic models. Front. Cell. Neurosci. 8:79521. doi: 10.3389/fncel.2014.00030 24570656 PMC3916725

[B59] Doll C. A. Broadie K. (2015). Activity-dependent FMRP requirements in development of the neural circuitry of learning and memory. Development 142, 1346–1356. doi: 10.1242/dev.117127 25804740 PMC4378248

[B60] Doll C. A. Broadie K. (2016). Neuron class-specific requirements for Fragile X mental retardation protein in critical period development of calcium signaling in learning and memory circuitry. Neurobiol. Dis. 89, 76–87. doi: 10.1016/j.nbd.2016.02.006 26851502 PMC4785039

[B61] Doll C. A. Vita D. J. Broadie K. (2017). Fragile X mental retardation protein requirements in activity-dependent critical period neural circuit refinement. Curr. Biol. 27, 2318–2330.e3. doi: 10.1016/j.cub.2017.06.046 28756946 PMC5572839

[B62] Dopp J. Ortega A. Davie K. Poovathingal S. Baz E. S. Liu S. (2024). Single-cell transcriptomics reveals that glial cells integrate homeostatic and circadian processes to drive sleep–wake cycles. Nat. Neurosci. 27, 359–372. doi: 10.1038/s41593-023-01549-4 38263460 PMC10849968

[B63] Dorkenwald S. Matsliah A. Sterling A. R. Schlegel P. Yu S. C. McKellar C. E. . (2024). Neuronal wiring diagram of an adult brain. Nature 634, 124–138. doi: 10.1038/s41586-024-07558-y 39358518 PMC11446842

[B64] Du Y. Miller V. K. Mellies A. J. Broadie K. (2026). Elevated serotonin receptor 2A signaling restores learning and memory in a Fragile X syndrome model. Sci. Rep. 16:4450. doi: 10.1038/s41598-025-34492-4 41501339 PMC12864886

[B65] Duan W. Cao D. Wang S. Cheng J. (2024). Serotonin 2A receptor (5-HT2AR) agonists: psychedelics and non-hallucinogenic analogues as emerging antidepressants. Chem. Rev. 124, 124–163. doi: 10.1021/acs.chemrev.3c00375 38033123

[B66] Duffy A. S. Eyo U. B. (2025). Microglia and astrocytes in postnatal neural circuit formation. Glia 73, 232–250. doi: 10.1002/glia.24650 39568399 PMC11662987

[B67] Duman R. S. Heninger G. R. Nestler E. J. (1997). A molecular and cellular theory of depression. Arch. Gen. Psychiatry 54, 597–606. doi: 10.1001/archpsyc.1997.01830190015002 9236543

[B68] Ebadi M. Simonneaux V. (1991). Ambivalence on the multiplicity of mammalian aromatic L-amino acid decarboxylase. Adv. Exp. Med. Biol. 294, 115–125. doi: 10.1007/978-1-4684-5952-4_10 1772061

[B69] Espinosa J. S. Stryker M. P. (2012). Development and plasticity of the primary visual cortex. Neuron 75:230. doi: 10.1016/j.neuron.2012.06.009 22841309 PMC3612584

[B70] Fabio M. C. Mujica M. V. Fogliatti E. Aguilar M. V. Degano A. L. Pautassi R. M. (2025). Prenatal serotonin depletion persistently disrupts social behavior and modulates ΔFosB and SERT expression in mice. Pharmacol. Biochem. Behav. 253:174043. doi: 10.1016/j.pbb.2025.174043 40451488

[B71] Farizatto K. L. G. Baldwin K. T. (2023). Astrocyte-synapse interactions during brain development. Curr. Opin. Neurobiol. 80:102704. doi: 10.1016/j.conb.2023.102704 36913751

[B72] Faust T. E. Gunner G. Schafer D. P. (2021). Mechanisms governing activity-dependent synaptic pruning in the developing mammalian CNS. Nat. Rev. Neurosci. 22, 657–673. doi: 10.1038/s41583-021-00507-y 34545240 PMC8541743

[B73] Faust T. E. Lee Y. H. O'Connor C. D. Boyle M. A. Gunner G. Durán-Laforet V. . (2025). Microglia-astrocyte crosstalk regulates synapse remodeling via Wnt signaling. Cell 188, 5212–5230.e21. doi: 10.1016/j.cell.2025.08.023 40934914 PMC12489809

[B74] Freeman M. R. (2015). *Drosophila* central nervous system glia. Cold Spring Harb. Perspect. Biol. 7:a020552. doi: 10.1101/cshperspect.a020552 25722465 PMC4632667

[B75] Froemke R. C. (2015). Plasticity of cortical excitatory-inhibitory balance. Annu. Rev. Neurosci. 38:195. doi: 10.1146/annurev-neuro-071714-034002 25897875 PMC4652600

[B76] Fuxe K. (1965). Evidence for the existence of monoamine neurons in the central nervous system. 3. The monoamine nerve terminal. Z. Zellforsch. Mikrosk. Anat. 65, 573–596. doi: 10.1007/BF00337069 14263017

[B77] Gajardo I. Guerra S. Campusano J. M. (2023). Navigating like a fly: *Drosophila* melanogaster as a model to explore the contribution of serotonergic neurotransmission to spatial navigation. Int. J. Mol. Sci. 24:4407. doi: 10.3390/ijms24054407 36901836 PMC10002024

[B78] Gaspar P. Cases O. Maroteaux L. (2003). The developmental role of serotonin: news from mouse molecular genetics. Nat. Rev. Neurosci. 4, 1002–1012. doi: 10.1038/nrn1256 14618156

[B79] Gerachshenko T. Blackmer T. Yoon E. J. Bartleson C. Hamm H. E. Alford S. (2005). Gbetagamma acts at the C terminus of SNAP-25 to mediate presynaptic inhibition. Nat. Neurosci. 8, 597–605. doi: 10.1038/nn1439 15834421

[B80] Glebov K. Löchner M. Jabs R. Lau T. Merkel O. Schloss P. . (2015). Serotonin stimulates secretion of exosomes from microglia cells. Glia 63, 626–634. doi: 10.1002/glia.22772 25451814

[B81] Gleichman A. J. Kawaguchi R. Sofroniew M. V. Carmichael S. T. (2023). A toolbox of astrocyte-specific, serotype-independent adeno-associated viral vectors using microRNA targeting sequences. Nat. Commun. 14:42746. doi: 10.1038/s41467-023-42746-w 37973910 PMC10654773

[B82] Golovin R. M. Broadie K. (2016). Developmental experience-dependent plasticity in the first synapse of the *Drosophila* olfactory circuit. J. Neurophysiol. 116, 2730–2738. doi: 10.1152/jn.00616.2016 27683892 PMC5133311

[B83] Golovin R. M. Broadie K. (2017). Neural circuits: reduced inhibition in Fragile X syndrome. Curr. Biol. 27, R298–R300. doi: 10.1016/j.cub.2017.03.011 28441561 PMC5734915

[B84] Golovin R. M. Vest J. Broadie K. (2021). Neuron-specific FMRP roles in experience-dependent remodeling of olfactory brain innervation during an early-life critical period. J. Neurosci. 41, 1218–1241. doi: 10.1523/JNEUROSCI.2167-20.2020 33402421 PMC7888229

[B85] Golovin R. M. Vest J. Vita D. J. Broadie K. (2019). Activity-dependent remodeling of *Drosophila* olfactory sensory neuron brain innervation during an early-life critical period. J. Neurosci. 39:2995. doi: 10.1523/JNEUROSCI.2223-18.2019 30755492 PMC6468095

[B86] González-Arias C. Sánchez-Ruiz A. Esparza J. Sánchez-Puelles C. Arancibia L. Ramírez-Franco J. . (2023). Dysfunctional serotonergic neuron-astrocyte signaling in depressive-like states. Mol. Psychiatry 28, 3856–3873. doi: 10.1038/s41380-023-02269-8 37773446 PMC10730416

[B87] Goodwin G. M. Aaronson S. T. Alvarez O. Arden P. C. Baker A. Bennett J. C. . (2022). Single-dose psilocybin for a treatment-resistant episode of major depression. N. Engl. J. Med. 387, 1637–1648. doi: 10.1056/NEJMoa2206443 36322843

[B88] Greiss Hess L. Fitzpatrick S. E. Nguyen D. V. Chen Y. Gaul K. N. Schneider A. . (2016). A randomized, double-blind, placebo-controlled trial of low-dose sertraline in young children with Fragile X syndrome. J. Dev. Behav. Pediatr. 37, 619–628. doi: 10.1097/DBP.0000000000000334 27560971 PMC5039060

[B89] Grieco S. F. Castrén E. Knudsen G. M. Kwan A. C. Olson D. E. Zuo Y. . (2022). Psychedelics and neural plasticity: therapeutic implications. J. Neurosci. 42, 8439–8449. doi: 10.1523/JNEUROSCI.1121-22.2022 36351821 PMC9665925

[B90] Gu N. Makashova O. Laporte C. Chen C. Q. Li B. Chevillard P. M. . (2025). Microglia regulate neuronal activity via structural remodeling of astrocytes. Neuron 113:107832. doi: 10.1016/j.neuron.2025.07.024 40834861

[B91] Guseva D. Wirth A. Ponimaskin E. (2014). Cellular mechanisms of the 5-HT7 receptor-mediated signaling. Front. Behav. Neurosci. 8:306. doi: 10.3389/fnbeh.2014.00306 25324743 PMC4181333

[B92] Hagihara H. Ohira K. Miyakawa T. (2019). Transcriptomic evidence for immaturity induced by antidepressant fluoxetine in the hippocampus and prefrontal cortex. Neuropsychopharmacol. Rep. 39, 78–89. doi: 10.1002/npr2.12048 30772953 PMC7292305

[B93] Hartenstein V. (2011). Morphological diversity and development of glia in *Drosophila*. Glia 59, 1237–1252. doi: 10.1002/glia.21162 21438012 PMC3950653

[B94] Heffel M. G. Zhou J. Zhang Y. Lee D. S. Hou K. Pastor-Alonso O. . (2024). Temporally distinct 3D multi-omic dynamics in the developing human brain. Nature 635, 481–489. doi: 10.1038/s41586-024-08030-7 39385032 PMC11560841

[B95] Hensch T. K. (2005). Critical period plasticity in local cortical circuits. Nat. Rev. Neurosci. 6, 877–888. doi: 10.1038/nrn1787 16261181

[B96] Herculano-Houzel S. (2009). The human brain in numbers: a linearly scaled-up primate brain. Front. Hum. Neurosci. 3:31. doi: 10.3389/neuro.09.031.2009 19915731 PMC2776484

[B97] Higa G. S. V. Francis-Oliveira J. Carlos-Lima E. Tamais A. M. Borges F. da S. . (2022). 5-HT-dependent synaptic plasticity of the prefrontal cortex in postnatal development. Sci. Rep. 12, 1–23. doi: 10.1038/s41598-022-23767-9 36470912 PMC9723183

[B98] Higa G. S. V. Viana F. J. C. Francis-Oliveira J. Cruvinel E. Franchin T. S. Marcourakis T. . (2024). Serotonergic neuromodulation of synaptic plasticity. Neuropharmacology 257:110036. doi: 10.1016/j.neuropharm.2024.110036 38876308

[B99] Hodes G. E. Hill-Smith T. E. Lucki I. (2010). Fluoxetine treatment induces dose dependent alterations in depression associated behavior and neural plasticity in female mice. Neurosci. Lett. 484, 12–16. doi: 10.1016/j.neulet.2010.07.084 20692322 PMC4623584

[B100] Hofto L. R. Lee C. E. Cafiero M. (2009). The importance of aromatic-type interactions in serotonin synthesis: protein-ligand interactions in tryptophan hydroxylase and aromatic amino acid decarboxylase. J. Comput. Chem. 30, 1111–1115. doi: 10.1002/jcc.21139 18942733

[B101] Holtmaat A. Svoboda K. (2009). Experience-dependent structural synaptic plasticity in the mammalian brain. Nat. Rev. Neurosci. 10, 647–658. doi: 10.1038/nrn2699 19693029

[B102] Hong S. Beja-Glasser V. F. Nfonoyim B. M. Frouin A. Li S. Ramakrishnan S. . (2016a). Complement and microglia mediate early synapse loss in Alzheimer mouse models. Science (1979) 352, 712–716. doi: 10.1126/science.aad8373 27033548 PMC5094372

[B103] Hong S. Dissing-Olesen L. Stevens B. (2016b). New insights on the role of microglia in synaptic pruning in health and disease. Curr. Opin. Neurobiol. 36, 128–134. doi: 10.1016/j.conb.2015.12.004 26745839 PMC5479435

[B104] Hordacre B. Austin D. Brown K. E. Graetz L. Pareés I. De Trane S. . (2021). Evidence for a window of enhanced plasticity in the human motor cortex following ischemic stroke. Neurorehabil. Neural Repair. 35, 307–320. doi: 10.1177/1545968321992330 33576318 PMC7610679

[B105] Hoyer D. Hannon J. P. Martin G. R. (2002). Molecular, pharmacological and functional diversity of 5-HT receptors. Pharmacol. Biochem. Behav. 71, 533–554. doi: 10.1016/S0091-3057(01)00746-8 11888546

[B106] Hoyer D. Martin G. (1997). 5-HT receptor classification and nomenclature: towards a harmonization with the human genome. Neuropharmacology 36, 419–428. doi: 10.1016/S0028-3908(97)00036-1 9225265

[B107] Hubel D. H. Wiesel T. N. (1970). The period of susceptibility to the physiological effects of unilateral eye closure in kittens. J. Physiol. 206, 419–436. doi: 10.1113/jphysiol.1970.sp009022 5498493 PMC1348655

[B108] Inserra A. De Gregorio D. Gobbi G. (2021). Psychedelics in psychiatry: neuroplastic, immunomodulatory, and neurotransmitter mechanisms. Pharmacol. Rev. 73, 202–277. doi: 10.1124/pharmrev.120.000056 33328244

[B109] Irwin S. A. Galvez R. Greenough W. T. (2000). Dendritic spine structural anomalies in fragile-X mental retardation syndrome. Cereb. Cortex 10, 1038–1044. doi: 10.1093/cercor/10.10.1038 11007554

[B110] Jacobs B. L. Fornal C. A. (1999). Activity of serotonergic neurons in behaving animals. Neuropsychopharmacology 21, 9S−15S. doi: 10.1038/sj.npp.1395336 10432483

[B111] Jiang Q. Shao L. X. Yao S. Savalia N. K. Gilbert A. D. Davoudian P. A. . (2026). Psilocybin triggers an activity-dependent rewiring of large-scale cortical networks. Cell 189, 659–675.e22. doi: 10.1016/j.cell.2025.11.009 41352354 PMC12695013

[B112] Jones K. A. Srivastava D. P. Allen J. A. Strachan R. T. Roth B. L. Penzes P. (2009). Rapid modulation of spine morphology by the 5-HT2A serotonin receptor through kalirin-7 signaling. Proc. Natl. Acad. Sci. U.S.A. 106, 19575–19580. doi: 10.1073/pnas.0905884106 19889983 PMC2780750

[B113] Kalambogias J. Chen C. C. Khan S. Son T. Wercberger R. Headlam C. . (2019). Development and sensory experience dependent regulation of microglia in barrel cortex. J. Comp. Neurol. 528:559. doi: 10.1002/cne.24771 31502243 PMC6944757

[B114] Kargbo R. B. (2024). Neuropharmacological advances: harnessing 5-HT2A receptor modulators and psychoplastogens. ACS Med. Chem. Lett. 15, 171–173. doi: 10.1021/acsmedchemlett.4c00003 38352827 PMC10860177

[B115] Katz L. C. Shatz C. J. (1996). Synaptic activity and the construction of cortical circuits. Science 274, 1133–1138. doi: 10.1126/science.274.5290.1133 8895456

[B116] Kenny B. J. Preuss C. V. Zito P. M. (2023). Controlled Substance Schedules. StatPearls. Available online at: https://www.ncbi.nlm.nih.gov/books/NBK538457/ (Accessed November 24, 2025). 30860707

[B117] Kim Y. Jeon S. J. Gonzales E. L. Shin D. Remonde C. G. Ahn T. . (2022). Pirenperone relieves the symptoms of fragile X syndrome in Fmr1 knockout mice. Sci. Rep. 12:20966. doi: 10.1038/s41598-022-25582-8 36470953 PMC9723111

[B118] Knudsen E. I. (1983). Early auditory experience aligns the auditory map of space in the optic tectum of the barn owl. Science 222, 939–942. doi: 10.1126/science.6635667 6635667

[B119] Kolodziejczak M. Béchade C. Gervasi N. Irinopoulou T. Banas S. M. Cordier C. . (2015). Serotonin modulates developmental microglia via 5-HT2B receptors: potential implication during synaptic refinement of retinogeniculate projections. ACS Chem. Neurosci. 6, 1219–1230. doi: 10.1021/cn5003489 25857335

[B120] Kristensen A. S. Andersen J. Jorgensen T. N. Sorensen L. Eriksen J. Loland C. J. . (2011). SLC6 neurotransmitter transporters: structure, function, and regulation. Pharmacol. Rev. 63, 585–640. doi: 10.1124/pr.108.000869 21752877

[B121] Kwon H. B. Sabatini B. L. (2011). Glutamate induces de novo growth of functional spines in developing cortex. Nature 474:100. doi: 10.1038/nature09986 21552280 PMC3107907

[B122] Larsen B. Sydnor V. J. Keller A. S. Yeo B. T. T. Satterthwaite T. D. (2023). A critical period plasticity framework for the sensorimotor–association axis of cortical neurodevelopment. Trends Neurosci. 46, 847–862. doi: 10.1016/j.tins.2023.07.007 37643932 PMC10530452

[B123] Lee J. Kim S. W. Kim K. T. (2022). Region-specific characteristics of astrocytes and microglia: a possible involvement in aging and diseases. Cells 11:1902. doi: 10.3390/cells11121902 35741031 PMC9220858

[B124] Lei W. Omotade O. F. Myers K. R. Zheng J. Q. (2016). Actin cytoskeleton in dendritic spine development and plasticity. Curr. Opin. Neurobiol. 39:86. doi: 10.1016/j.conb.2016.04.010 27138585 PMC4987222

[B125] Lewis V. Bonniwell E. M. Lanham J. K. Ghaffari A. Sheshbaradaran H. Cao A. B. . (2023). A non-hallucinogenic LSD analog with therapeutic potential for mood disorders. Cell Rep. 42:112203. doi: 10.1016/j.celrep.2023.112203 36884348 PMC10112881

[B126] Li W. Ma L. Yang G. Gan W. B. (2017). REM sleep selectively prunes and maintains new synapses in development and learning. Nat. Neurosci. 20, 427–437. doi: 10.1038/nn.4479 28092659 PMC5535798

[B127] Ligneul R. Mainen Z. F. (2023). Serotonin. Curr. Biol. 33, R1216–R1221. doi: 10.1016/j.cub.2023.09.068 38052167

[B128] Liu H. Leak R. K. Hu X. (2016). Neurotransmitter receptors on microglia. Stroke Vasc. Neurol. 1, 52–58. doi: 10.1136/svn-2016-000012 28959464 PMC5435193

[B129] Liu J. Niethard N. Lun Y. Dimitrov S. Ehrlich I. Born J. . (2024). Slow-wave sleep drives sleep-dependent renormalization of synaptic AMPA receptor levels in the hypothalamus. PLoS Biol. 22:e3002768. doi: 10.1371/journal.pbio.3002768 39163472 PMC11364421

[B130] Liu X. Kumar V. Tsai N.-P. Auerbach B. D. (2021). Hyperexcitability and homeostasis in Fragile X syndrome. Front. Mol. Neurosci. 14:805929. doi: 10.3389/fnmol.2021.805929 35069112 PMC8770333

[B131] López-Giménez J. F. González-Maeso J. (2018). Hallucinogens and serotonin 5-HT2A receptor-mediated signaling pathways. Curr. Top. Behav. Neurosci. 36:45. doi: 10.1007/7854_2017_478 28677096 PMC5756147

[B132] Ludwig P. E. Das J. M. (2023). Histology, Glial Cells. StatPearls. Available online at: https://www.ncbi.nlm.nih.gov/books/NBK441945/ (Accessed November 24, 2025).

[B133] Ly C. Greb A. C. Cameron L. P. Wong J. M. Barragan E. V. Wilson P. C. . (2018). Psychedelics promote structural and functional neural plasticity. Cell Rep. 23:3170. doi: 10.1016/j.celrep.2018.05.022 29898390 PMC6082376

[B134] Lyon K. A. Allen N. J. (2022). From synapses to circuits, astrocytes regulate behavior. Front. Neural Circuits 15:786293. doi: 10.3389/fncir.2021.786293 35069124 PMC8772456

[B135] MacDonald J. M. Beach M. G. Porpiglia E. Sheehan A. E. Watts R. J. Freeman M. R. (2006). The *Drosophila* cell corpse engulfment receptor Draper mediates glial clearance of severed axons. Neuron 50, 869–881. doi: 10.1016/j.neuron.2006.04.028 16772169

[B136] Macnamee S. E. Liu K. E. Gerhard S. Tran C. T. Fetter R. D. Cardona A. . (2016). Astrocytic glutamate transport regulates a *Drosophila* CNS synapse that lacks astrocyte ensheathment. J. Comp. Neurol. 524, 1979–1998. doi: 10.1002/cne.24016 27073064 PMC4861170

[B137] Malberg J. E. Eisch A. J. Nestler E. J. Duman R. S. (2000). Chronic antidepressant treatment increases neurogenesis in adult rat hippocampus. J. Neurosci. 20, 9104–9110. doi: 10.1523/JNEUROSCI.20-24-09104.2000 11124987 PMC6773038

[B138] Marciante A. B. Tadjalli A. Nikodemova M. Burrowes K. A. Oberto J. Luca E. K. . (2024). Microglia regulate motor neuron plasticity via reciprocal fractalkine and adenosine signaling. Nat. Commun. 15:54619. doi: 10.1038/s41467-024-54619-x 39609435 PMC11605081

[B139] Masson J. Emerit M. B. Hamon M. Darmon M. (2012). Serotonergic signaling: multiple effectors and pleiotropic effects. Wiley Interdiscip. Rev. Membr. Transp. Signal. 1, 685–713. doi: 10.1002/wmts.50

[B140] Mcisaac W. M. Page I. H. (1959). The metabolism of serotonin (5-hydroxytryptamine). J. Biol. Chem. 234, 858–864. doi: 10.1016/S0021-9258(18)70190-7 13654278

[B141] Mi Z. Si T. Kapadia K. Li Q. Muma N. A. (2017). Receptor-stimulated transamidation induces activation of Rac1 and Cdc42 and the regulation of dendritic spines. Neuropharmacology 117:93. doi: 10.1016/j.neuropharm.2017.01.034 28161375 PMC5386786

[B142] Miceli S. Negwer M. van Eijs F. Kalkhoven C. van Lierop I. Homberg J. . (2013). High serotonin levels during brain development alter the structural input-output connectivity of neural networks in the rat somatosensory layer IV. Front. Cell. Neurosci. 7:88. doi: 10.3389/fncel.2013.00088 23761736 PMC3675331

[B143] Miller V. K. Broadie K. (2024). Experience-dependent serotonergic signaling in glia regulates targeted synapse elimination. PLoS Biol. 22:e3002822. doi: 10.1371/journal.pbio.3002822 39352884 PMC11444420

[B144] Miller V. K. Broadie K. (2025). Glia-to-glia serotonin signaling directs MMP-dependent infiltration for experience-dependent synapse pruning. PLoS Biol. 23:e3003524. doi: 10.1371/journal.pbio.3003524 41325349 PMC12668486

[B145] Mitchell J. M. Bogenschutz M. Lilienstein A. Harrison C. Kleiman S. Parker-Guilbert K. . (2021). MDMA-assisted therapy for severe PTSD: a randomized, double-blind, placebo-controlled phase 3 study. Nat. Med. 27, 1025–1033. doi: 10.1038/s41591-021-01336-3 33972795 PMC8205851

[B146] Moliner R. Girych M. Brunello C. A. Kovaleva V. Biojone C. Enkavi G. . (2023). Psychedelics promote plasticity by directly binding to BDNF receptor TrkB. Nat. Neurosci. 2023 26:6 26, 1032–1041. doi: 10.1038/s41593-023-01316-5 37280397 PMC10244169

[B147] Monti J. M. (2011). Serotonin control of sleep-wake behavior. Sleep Med. Rev. 15, 269–281. doi: 10.1016/j.smrv.2010.11.003 21459634

[B148] Mortimer K. R. H. Katshu M. Z. U. H. Chakrabarti L. (2023). Second-generation antipsychotics and metabolic syndrome: a role for mitochondria. Front. Psychiatry 14:1257460. doi: 10.3389/fpsyt.2023.1257460 38076704 PMC10704249

[B149] Mukhopadhyay S. Chatterjee A. Tiwari P. Ghai U. Vaidya V. A. (2021). Postnatal fluoxetine treatment alters perineuronal net formation and maintenance in the hippocampus. eNeuro 8:ENEURO.0424-20.2021. doi: 10.1523/ENEURO.0424-20.2021 33622703 PMC8046023

[B150] Müller F. E. Schade S. K. Cherkas V. Stopper L. Breithausen B. Minge D. . (2021). Serotonin receptor 4 regulates hippocampal astrocyte morphology and function. Glia 69, 872–889. doi: 10.1002/glia.23933 33156956

[B151] Nardou R. Sawyer E. Song Y. J. Wilkinson M. Padovan-Hernandez Y. de Deus J. L. . (2023). Psychedelics reopen the social reward learning critical period. Nature 618, 790–798. doi: 10.1038/s41586-023-06204-3 37316665 PMC10284704

[B152] Neckameyer W. S. Coleman C. M. Eadie S. Goodwin S. F. (2007). Compartmentalization of neuronal and peripheral serotonin synthesis in *Drosophila* melanogaster. Genes Brain Behav. 6, 756–769. doi: 10.1111/j.1601-183X.2007.00307.x 17376153

[B153] Nelson N. Broadie K. (2025). Neuron-to-glia signaling drives critical period experience-dependent synapse pruning. Sci. Rep. 15:25744. doi: 10.1038/s41598-025-11528-3 40670566 PMC12267418

[B154] Nelson N. Miller V. Broadie K. (2025). Neuron-to-glia and glia-to-glia signaling directs critical period experience-dependent synapse pruning. Front. Cell Dev. Biol. 13:1540052. doi: 10.3389/fcell.2025.1540052 40040788 PMC11876149

[B155] Nelson N. Vita D. J. Broadie K. (2024). Experience-dependent glial pruning of synaptic glomeruli during the critical period. Sci. Rep. 14:9110. doi: 10.1038/s41598-024-59942-3 38643298 PMC11032375

[B156] Neniskyte U. Gross C. T. (2017). Errant gardeners: glial-cell-dependent synaptic pruning and neurodevelopmental disorders. Nat. Rev. Neurosci. 18, 658–670. doi: 10.1038/nrn.2017.110 28931944

[B157] Nguyen P. T. Dorman L. C. Pan S. Vainchtein I. D. Han R. T. Nakao-Inoue H. . (2020). Microglial remodeling of the extracellular matrix promotes synapse plasticity. Cell 182, 388–403.e15. doi: 10.1016/j.cell.2020.05.050 32615087 PMC7497728

[B158] Ni R. J. Yuan W. J. Wang Y. Y. Yang X. Wei J. X. Zhao L. S. . (2025). Microglia-mediated inflammation and synaptic pruning contribute to sleep deprivation-induced mania in a sex-specific manner. Transl. Psychiatry 15:249. doi: 10.1038/s41398-025-03525-x 40817224 PMC12356969

[B159] Nichols D. E. (2016). Psychedelics. Pharmacol. Rev. 68:264. doi: 10.1124/pr.115.011478 26841800 PMC4813425

[B160] Noguchi T. Nishino M. Kido R. (1973). Tryptophan 5-hydroxylase in rat intestine. Biochem. J. 131, 375–380. doi: 10.1042/bj1310375 4541815 PMC1177478

[B161] Ocana-Santero G. Warming H. Munday V. MacKay H. A. Gibeily C. Hemingway C. . (2025). Perinatal serotonin signalling dynamically influences the development of cortical GABAergic circuits with consequences for lifelong sensory encoding. Nat. Commun. 16:59659. doi: 10.1038/s41467-025-59659-5 40467568 PMC12137630

[B162] Ogelman R. Gomez Wulschner L. E. Hoelscher V. M. Hwang I. W. Chang V. N. Oh W. C. (2024). Serotonin modulates excitatory synapse maturation in the developing prefrontal cortex. Nat. Commun. 15:45734. doi: 10.1038/s41467-024-45734-w 38365905 PMC10873381

[B163] Ohtani A. Kozono N. Senzaki K. Shiga T. (2014). Serotonin 2A receptor regulates microtubule assembly and induces dynamics of dendritic growth cones in rat cortical neurons in vitro. Neurosci. Res. 81–82, 11–20. doi: 10.1016/j.neures.2014.03.006 24698813

[B164] O'Leary O. F. Wu X. Castren E. (2009). Chronic fluoxetine treatment increases expression of synaptic proteins in the hippocampus of the ovariectomized rat: role of BDNF signalling. Psychoneuroendocrinology 34, 367–381. doi: 10.1016/j.psyneuen.2008.09.015 18977602

[B165] Omoto J. J. Yogi P. Hartenstein V. (2015). Origin and development of neuropil glia of the *Drosophila* larval and adult brain: two distinct glial populations derived from separate progenitors. Dev. Biol. 404:2. doi: 10.1016/j.ydbio.2015.03.004 25779704 PMC4515183

[B166] Osmond H. (1957). A review of the clinical effects of psychotomimetic agents. Ann. N.Y. Acad. Sci. 66, 418–434. doi: 10.1111/j.1749-6632.1957.tb40738.x 13425232

[B167] Ota Y. Zanetti A. T. Hallock R. M. (2013). The Role of Astrocytes in the Regulation of Synaptic Plasticity and Memory Formation. Neural Plast. 2013, 185463. doi: 10.1155/2013/185463 24369508 PMC3867861

[B168] Özçete Ö. D. Banerjee A. Kaeser P. S. (2024). Mechanisms of neuromodulatory volume transmission. Molecular Psychiatry 2024 29:11 29, 3680–3693. doi: 10.1038/s41380-024-02608-3 38789677 PMC11540752

[B169] Pan L. Zhang Y. Q. Woodruff E. Broadie K. (2004). The *Drosophila* fragile X gene negatively regulates neuronal elaboration and synaptic differentiation. Curr. Biol. 14, 1863–1870. doi: 10.1016/j.cub.2004.09.085 15498496

[B170] Paolicelli R. C. Bolasco G. Pagani F. Maggi L. Scianni M. Panzanelli P. . (2011). Synaptic pruning by microglia is necessary for normal brain development. Science 333, 1456–1458. doi: 10.1126/science.1202529 21778362

[B171] Parkins S. Song Y. Jaoui Y. Gala A. Konda K. T. Richardson C. . (2025). Spatial mapping of activity changes across sensory areas following visual deprivation in adults. J. Neurosci. 45. doi: 10.1523/JNEUROSCI.0969-24.2024 39592237 PMC11756622

[B172] Parpura V. Basarsky T. A. Liu F. Jeftinija K. Jeftinija S. Haydon P. G. (1994). Glutamate-mediated astrocyte-neuron signalling. Nature 369, 744–747. doi: 10.1038/369744a0 7911978

[B173] Patel T. D. Zhou F. C. (2005). Ontogeny of 5-HT1A receptor expression in the developing hippocampus. Brain Res. Dev. Brain Res. 157, 42–57. doi: 10.1016/j.devbrainres.2005.03.006 15939084

[B174] Patton M. H. Blundon J. A. Zakharenko S. S. (2019). Rejuvenation of plasticity in the brain: opening the critical period. Curr. Opin. Neurobiol. 54, 83–89. doi: 10.1016/j.conb.2018.09.003 30286407 PMC6361689

[B175] Penzes P. Rafalovich I. (2012). Regulation of the actin cytoskeleton in dendritic spines. Adv. Exp. Med. Biol. 970, 81–95. doi: 10.1007/978-3-7091-0932-8_4 22351052 PMC3576144

[B176] Peter R. H. (1979). Synaptic density in human frontal cortex - Developmental changes and effects of aging. Brain Res. 163, 195–205. doi: 10.1016/0006-8993(79)90349-4 427544

[B177] Pizzorusso T. Medini P. Berardi N. Chierzi S. Fawcett J. W. Maffei L. (2002). Reactivation of ocular dominance plasticity in the adult visual cortex. Science 298, 1248–1251. doi: 10.1126/science.1072699 12424383

[B178] Pons T. P. Garraghty P. E. Ommaya A. K. Kaas J. H. Taub E. Mishkin M. (1991). Massive cortical reorganization after sensory deafferentation in adult macaques. Science 252, 1857–1860. doi: 10.1126/science.1843843 1843843

[B179] Popa D. Léna C. Fabre V. Prenat C. Gingrich J. Escourrou P. . (2005). Contribution of 5-HT2 receptor subtypes to sleep-wakefulness and respiratory control, and functional adaptations in knock-out mice lacking 5-HT2A receptors. J. Neurosci. 25, 11231–11238. doi: 10.1523/JNEUROSCI.1724-05.2005 16339018 PMC6725907

[B180] Pourhamzeh M. Moravej F. G. Arabi M. Shahriari E. Mehrabi S. Ward R. . (2021). The roles of serotonin in neuropsychiatric disorders. Cell. Mol. Neurobiol. 42:1671. doi: 10.1007/s10571-021-01064-9 33651238 PMC11421740

[B181] Raiders S. Han T. Scott-Hewitt N. Kucenas S. Lew D. Logan M. A. . (2021). Engulfed by glia: glial pruning in development, function, and injury across species. J. Neurosci. 41, 823–833. doi: 10.1523/JNEUROSCI.1660-20.2020 33468571 PMC7880271

[B182] Raison C. L. Sanacora G. Woolley J. Heinzerling K. Dunlop B. W. Brown R. T. . (2023). Single-dose psilocybin treatment for major depressive disorder: a randomized clinical trial. JAMA 330, 843–853. doi: 10.1001/jama.2023.14530 37651119 PMC10472268

[B183] Rebello T. J. Yu Q. Caffrey Cagliostro M. K. Teissier A. Morelli E. Demireva E. Y. . (2014). Postnatal day 2 to 11 constitutes a 5-HT-sensitive period impacting adult mPFC function. J. Neurosci. 34:12379. doi: 10.1523/JNEUROSCI.1020-13.2014 25209278 PMC4160773

[B184] Reha R. K. Dias B. G. Nelson C. A. Kaufer D. Werker J. F. Kolbh B. . (2020). Critical period regulation across multiple timescales. Proc. Natl. Acad. Sci. U.S.A. 117, 23242–23251. doi: 10.1073/pnas.1820836117 32503914 PMC7519216

[B185] Ribot J. Breton R. Calvo C. F. Moulard J. Ezan P. Zapata J. . (2021). Astrocytes close the mouse critical period for visual plasticity. Science 373, 77–81. doi: 10.1126/science.abf5273 34210880

[B186] Rojas P. S. Aguayo F. Neira D. Tejos M. Aliaga E. Muñoz J. P. . (2017). Dual effect of serotonin on the dendritic growth of cultured hippocampal neurons: involvement of 5-HT1A and 5-HT7 receptors. Mol. Cell. Neurosci. 85, 148–161. doi: 10.1016/j.mcn.2017.09.009 28974382

[B187] Rosenfeld C. S. (2019). Placental serotonin signaling, pregnancy outcomes, and regulation of fetal brain development†. Biol. Reprod. 102:532. doi: 10.1093/biolre/ioz204 31711155 PMC7443348

[B188] Sachs B. D. Caron M. G. (2014). Chronic fluoxetine increases extra-hippocampal neurogenesis in adult mice. Int. J. Neuropsychopharmacol. 18, 1–12. doi: 10.1093/ijnp/pyu029 25583694 PMC4360216

[B189] Saeger H. N. Olson D. E. (2022). Psychedelic-inspired approaches for treating neurodegenerative disorders. J. Neurochem. 162, 109–127. doi: 10.1111/jnc.15544 34816433 PMC9126991

[B190] Salerno C. Borri Voltattorni C. Giartosio A. Fiori A. Turano C. (1984). Kinetics of the reaction of DOPA decarboxylase with 5-hydroxy-L-tryptophan. Prog. Clin. Biol. Res. 144, 277–287. 6610180

[B191] Schaefer T. L. Davenport M. H. Erickson C. A. (2015). Emerging pharmacologic treatment options for fragile X syndrome. Appl. Clin. Genet. 8:75. doi: 10.2147/TACG.S35673 25897255 PMC4396424

[B192] Schafer D. P. Lehrman E. K. Kautzman A. G. Koyama R. Mardinly A. R. Yamasaki R. . (2012). Microglia sculpt postnatal neural circuits in an activity and complement-dependent manner. Neuron 74, 691–705. doi: 10.1016/j.neuron.2012.03.026 22632727 PMC3528177

[B193] Schafer D. P. Lehrman E. K. Stevens B. (2013). The “quad-partite” synapse: microglia-synapse interactions in the developing and mature CNS. Glia 61, 24–36. doi: 10.1002/glia.22389 22829357 PMC4082974

[B194] Schlegel P. Yin Y. Bates A. S. Dorkenwald S. Eichler K. Brooks P. . (2024). Whole-brain annotation and multi-connectome cell typing of *Drosophila*. Nature 634, 139–152. doi: 10.1038/s41586-024-07686-5 39358521 PMC11446831

[B195] Scott-Hewitt N. Perrucci F. Morini R. Erreni M. Mahoney M. Witkowska A. . (2020). Local externalization of phosphatidylserine mediates developmental synaptic pruning by microglia. EMBO J. 39:e105380. doi: 10.15252/embj.2020105380 32657463 PMC7429741

[B196] Sears J. C. Broadie K. (2018). Fragile X mental retardation protein regulates activity-dependent membrane trafficking and trans-synaptic signaling mediating synaptic remodeling. Front. Mol. Neurosci 10:440. doi: 10.3389/fnmol.2017.00440 29375303 PMC5770364

[B197] Sellgren C. M. Gracias J. Watmuff B. Biag J. D. Thanos J. M. Whittredge P. B. . (2019). Increased synapse elimination by microglia in schizophrenia patient-derived models of synaptic pruning. Nat. Neurosci. 22, 374–385. doi: 10.1038/s41593-018-0334-7 30718903 PMC6410571

[B198] Shan L. Zhang T. Fan K. Cai W. Liu H. (2021). Astrocyte-neuron signaling in synaptogenesis. Front. Cell Dev. Biol. 9, 680301–680301. doi: 10.3389/fcell.2021.680301 34277621 PMC8284252

[B199] Shang Z. Zhang X. (2025). Postnatal critical-period brain plasticity and neurodevelopmental disorders: revisited circuit mechanisms. J. Genet. Genom. 52:101613. doi: 10.1016/j.jgg.2025.07.006 40691895

[B200] Shao L. X. Liao C. Davoudian P. A. Savalia N. K. Jiang Q. Wojtasiewicz C. . (2025). Psilocybin's lasting action requires pyramidal cell types and 5-HT2A receptors. Nature 642, 411–420. doi: 10.1038/s41586-025-08813-6 40175553 PMC12188471

[B201] Sharp T. Ippolito A. (2025). Neuropsychopharmacology of hallucinogenic and non-hallucinogenic 5-HT2A receptor agonists. Br. J. Pharmacol. 182:70050. doi: 10.1111/bph.70050 40405723

[B202] Sitaraman D. Zars M. LaFerriere H. Chen Y. C. Sable-Smith A. Kitamoto T. . (2008). Serotonin is necessary for place memory in *Drosophila*. Proc. Natl. Acad. Sci. U.S.A. 105:5579. doi: 10.1073/pnas.0710168105 18385379 PMC2291120

[B203] Slifirski G. Król M. Turło J. (2021). 5-HT receptors and the development of new antidepressants. Int. J. Mol. Sci. 22:9015. doi: 10.3390/ijms22169015 34445721 PMC8396477

[B204] Sneddon J. M. (1973). Blood platelets as a model for monoamine-containing neurones. Prog. Neurobiol. 1, 151–198. doi: 10.1016/0301-0082(73)90019-1 4273118

[B205] Song C. Broadie K. (2022). Dysregulation of BMP, Wnt, and insulin signaling in Fragile X syndrome. Front. Cell Dev. Biol. 10:934662. doi: 10.3389/fcell.2022.934662 35880195 PMC9307498

[B206] Song C. Broadie K. (2023). Fragile X mental retardation protein coordinates neuron-to-glia communication for clearance of developmentally transient brain neurons. Proc. Natl. Acad. Sci. U.S.A. 120:e2216887120. doi: 10.1073/pnas.2216887120 36920921 PMC10041173

[B207] Sugie A. Hakeda-Suzuki S. Suzuki E. Silies M. Shimozono M. Möhl C. . (2015). Molecular remodeling of the presynaptic active zone of *Drosophila* photoreceptors via activity-dependent feedback. Neuron 86, 711–725. doi: 10.1016/j.neuron.2015.03.046 25892303

[B208] Sundström E. Kölare S. Souverbic F. Samuelsson E. B. Pschera H. Lunell N. O. . (1993). Neurochemical differentiation of human bulbospinal monoaminergic neurons during the first trimester. Brain Res. Dev. Brain Res. 75, 1–12. doi: 10.1016/0165-3806(93)90059-J 7900931

[B209] Suzuki Y. Schenk J. E. Tan H. Gaudry Q. (2020). A population of interneurons signals changes in the basal concentration of serotonin and mediates gain control in the *Drosophila* antennal lobe. Curr. Biol. 30, 1110–1118.e4. doi: 10.1016/j.cub.2020.01.018 32142699 PMC7133499

[B210] Sykes P. A. Condron B. G. (2005). Development and sensitivity to serotonin of *Drosophila* serotonergic varicosities in the central nervous system. Dev. Biol. 286:207. doi: 10.1016/j.ydbio.2005.07.025 16122730 PMC2896038

[B211] Takesian A. E. Bogart L. J. Lichtman J. W. Hensch T. K. (2018). Inhibitory circuit gating of auditory critical period plasticity. Nat. Neurosci. 21:218. doi: 10.1038/s41593-017-0064-2 29358666 PMC5978727

[B212] Tamir H. Liu K. P. Adlersberg M. Hsiung S. C. Gershon M. D. (1996). Acidification of serotonin-containing secretory vesicles induced by a plasma membrane calcium receptor. J. Biol. Chem. 271, 6441–6450. doi: 10.1074/jbc.271.11.6441 8626445

[B213] Tessier C. R. Broadie K. (2008). *Drosophila* fragile X mental retardation protein developmentally regulates activity-dependent axon pruning. Development 135, 1547–1557. doi: 10.1242/dev.015867 18321984 PMC3988902

[B214] Tewari B. P. Chaunsali L. Prim C. E. Sontheimer H. (2022). A glial perspective on the extracellular matrix and perineuronal net remodeling in the central nervous system. Front. Cell. Neurosci. 16:1022754. doi: 10.3389/fncel.2022.1022754 36339816 PMC9630365

[B215] Tononi G. Cirelli C. (2006). Sleep function and synaptic homeostasis. Sleep Med. Rev. 10, 49–62. doi: 10.1016/j.smrv.2005.05.002 16376591

[B216] Törk I. (1990). Anatomy of the serotonergic system. Ann. N.Y. Acad. Sci. 600, 9–34. doi: 10.1111/j.1749-6632.1990.tb16870.x 2252340

[B217] Tyler W. J. Murthy V. N. (2004). Synaptic vesicles. Curr. Biol. 14, R294–R297. doi: 10.1016/j.cub.2004.03.046 15084295

[B218] Udoh U. G. Bruno J. R. Osborn P. O. Pratt K. G. (2024). Serotonin strengthens a developing glutamatergic synapse through a PI3K-dependent mechanism. J. Neurosci. 44. doi: 10.1523/JNEUROSCI.1260-23.2023 38169457 PMC10860612

[B219] Ursin R. Bjorvatn B. Sommerfelt L. Underland G. (1989). Increased waking as well as increased synchronization following administration of selective 5-HT uptake inhibitors to rats. Behav. Brain Res. 34, 117–130. doi: 10.1016/S0166-4328(89)80095-6 2527519

[B220] Vanrobaeys Y. Peterson Z. J. Walsh E. N. Chatterjee S. Lin L. C. Lyons L. C. . (2023). Spatial transcriptomics reveals unique gene expression changes in different brain regions after sleep deprivation. Nat. Commun. 14:42751. doi: 10.1038/s41467-023-42751-z 37925446 PMC10625558

[B221] Vargas M. V. Dunlap L. E. Dong C. Carter S. J. Tombari R. J. Jami S. A. . (2023). Psychedelics promote neuroplasticity through the activation of intracellular 5-HT2A receptors. Science 379, 700–706. doi: 10.1126/science.adf0435 36795823 PMC10108900

[B222] Vecino E. Rodriguez F. D. Ruzafa N. Pereiro X. Sharma S. C. (2016). Glia–neuron interactions in the mammalian retina. Prog. Retin. Eye Res. 51, 1–40. doi: 10.1016/j.preteyeres.2015.06.003 26113209

[B223] Verkhratsky A. Parpura V. Scuderi C. Li B. (2021). Astroglial serotonin receptors as the central target of classic antidepressants. Adv. Neurobiol. 26:317. doi: 10.1007/978-3-030-77375-5_13 34888840 PMC9015684

[B224] Vicenzi S. Foa L. Gasperini R. J. (2020). Serotonin functions as a bidirectional guidance molecule regulating growth cone motility. Cell. Mol. Life Sci. 78:2247. doi: 10.1007/s00018-020-03628-2 32939562 PMC11072016

[B225] Vilalta A. Brown G. C. (2018). Neurophagy, the phagocytosis of live neurons and synapses by glia, contributes to brain development and disease. FEBS J. 285, 3566–3575. doi: 10.1111/febs.14323 29125686

[B226] Vinogradov S. Chafee M. V. Lee E. Morishita H. (2023). Psychosis spectrum illnesses as disorders of prefrontal critical period plasticity. Neuropsychopharmacology 48, 168–185. doi: 10.1038/s41386-022-01451-w 36180784 PMC9700720

[B227] Vita D. J. Meier C. J. Broadie K. (2021). Neuronal fragile X mental retardation protein activates glial insulin receptor mediated PDF-Tri neuron developmental clearance. Nat. Commun. 12:1160. doi: 10.1038/s41467-021-21429-4 33608547 PMC7896095

[B228] von Bartheld C. S. Bahney J. Herculano-Houzel S. (2016). The search for true numbers of neurons and glial cells in the human brain: a review of 150 years of cell counting. J. Comp. Neurol. 524, 3865–3895. doi: 10.1002/cne.24040 27187682 PMC5063692

[B229] Wagor E. Mangini N. J. Pearlman A. L. (1980). Retinotopic organization of striate and extrastriate visual cortex in the mouse. J. Comp. Neurol. 193, 187–202. doi: 10.1002/cne.901930113 6776164

[B230] Wallach J. Cao A. B. Calkins M. M. Heim A. J. Lanham J. K. Bonniwell E. M. . (2023). Identification of 5-HT2A receptor signaling pathways associated with psychedelic potential. Nat. Commun. 14:44016. doi: 10.1038/s41467-023-44016-1 38102107 PMC10724237

[B231] Wang F. Xu Q. Wang W. Takano T. Nedergaard M. (2012). Bergmann glia modulate cerebellar Purkinje cell bistability via Ca2+-dependent K+ uptake. Proc. Natl. Acad. Sci. U.S.A. 109, 7911–7916. doi: 10.1073/pnas.1120380109 22547829 PMC3356677

[B232] Wang J. W. David D. J. Monckton J. E. Battaglia F. Hen R. (2008). Chronic fluoxetine stimulates maturation and synaptic plasticity of adult-born hippocampal granule cells. J. Neurosci. 28, 1374–1384. doi: 10.1523/JNEUROSCI.3632-07.2008 18256257 PMC6671574

[B233] Wang L. Ling H. He H. Hu N. Xiao L. Zhang Y. . (2023). Dysfunctional synaptic pruning by microglia correlates with cognitive impairment in sleep-deprived mice: involvement of CX3CR1 signaling. Neurobiol. Stress 25:100553. doi: 10.1016/j.ynstr.2023.100553 37547773 PMC10401339

[B234] Wark C. Galliher J. F. (2010). Timothy Leary, Richard Alpert (Ram Dass) and the changing definition of psilocybin. Int. J. Drug Policy 21, 234–239. doi: 10.1016/j.drugpo.2009.08.004 19744846

[B235] Wegiel J. Chadman K. London E. Wisniewski T. Wegiel J. (2024). Contribution of the serotonergic system to developmental brain abnormalities in autism spectrum disorder. Autism Res. 17:1300. doi: 10.1002/aur.3123 38500252 PMC11272444

[B236] Weiss F. Magnesa A. Gambini M. Gurrieri R. Annuzzi E. Elefante C. . (2025). Psychedelic-induced neural plasticity: a comprehensive review and a discussion of clinical implications. Brain Sci. 15:117. doi: 10.3390/brainsci15020117 40002450 PMC11853016

[B237] Whitaker-Azmitia P. M. (2001). Serotonin and brain development: role in human developmental diseases. Brain Res. Bull. 56, 479–485. doi: 10.1016/S0361-9230(01)00615-3 11750793

[B238] Whitaker-Azmitia P. M. Shemer A. V. Caruso J. Molino L. Azmitia E. C. (1990). Role of high affinity serotonin receptors in neuronal growth. Ann. N.Y. Acad. Sci. 600, 315–330. doi: 10.1111/j.1749-6632.1990.tb16892.x 2252318

[B239] Wilkinson K. El Khoury J. (2012). Microglial scavenger receptors and their roles in the pathogenesis of Alzheimer's disease. Int. J. Alzheimers Dis. 2012:489456. doi: 10.1155/2012/489456 22666621 PMC3362056

[B240] Woolley D. W. (1958). Serotonin in mental disorders. Res. Publ. Assoc. Res. Nerv. Ment. Dis. 36, 381–400. 13527794

[B241] Wu X. Liu Y. Wang X. Zheng L. Pan L. Wang H. (2024). Developmental impairments of synaptic refinement in the thalamus of a mouse model of Fragile X syndrome. Neurosci. Bull. 40, 439–450. doi: 10.1007/s12264-023-01142-6 38015349 PMC11004103

[B242] Yekehtaz H. Farokhnia M. Akhondzadeh S. (2013). Cardiovascular considerations in antidepressant therapy: an evidence-based review. J. Tehran Univ. Heart Center 8:169. 26005484 PMC4434967

[B243] Zhao X. Ozols A. B. Meyers K. T. Campbell J. McBride A. Marballi K. K. . (2022). Acute sleep deprivation upregulates serotonin 2A receptors in the frontal cortex of mice via the immediate early gene Egr3. Mol. Psychiatry 27, 1599–1610. doi: 10.1038/s41380-021-01390-w 35001075 PMC9210263

[B244] Zheng Y. Xu L. (2025). Bidirectional crosstalk between microglia and serotonin signaling in neuroinflammation and CNS disorders. Front. Immunol. 16:1646740. doi: 10.3389/fimmu.2025.1646740 40934003 PMC12417188

[B245] Zuo X. Zhu Z. K. Liu M. Y. Zhao Q. Li X. Y. Zhao X. . (2024). Fluoxetine ameliorates cognitive deficits in high-fat diet mice by regulating BDNF expression. ACS Chem. Neurosci. 15, 4229–4240. doi: 10.1021/acschemneuro.4c00540 39476817

[B246] Zuo Y. Yang G. Kwon E. Gan W. B. (2005). Long-term sensory deprivation prevents dendritic spine loss in primary somatosensory cortex. Nature 436, 261–265. doi: 10.1038/nature03715 16015331

